# Supplementary material for: Phylogenomics reveals the history of host use in mosquitoes
Source: Nat Commun. 2023 Oct 6;14:6252. doi: 10.1038/s41467-023-41764-y (PMC10558525; doi:10.1038/s41467-023-41764-y)
Supplement: Supplementary file 1 — Supplementary Information [file 41467_2023_41764_MOESM1_ESM.pdf]

Soghigian *et al.* Supplementary Information

Table of Contents

I. Supplementary Discussion 1
1. A Time Tree for Mosquitoes....1
2. The Evolution of Host Associations in Mosquitoes 2
3. Systematics of the Culicidae....4
II. Supplementary Notes 7
1. Supplementary Note 1....7
Taxon Sampling, Quality Assessment, and Dataset Characteristics....7
2. Supplementary Note 2....7
Topological Congruency Across Analyses and the Influence of Saturation 7
3. Supplementary Note 3....8
Clock Selection, Divergence Time Estimation, and Convergence of Chains....8
4. Supplementary Note 4....9
Database of Host Associations....9
5. Supplementary Note 5....9
Phylogenetic Comparative Methods....9
III. Supplementary References....1
IV. SUPPLEMENTARY TABLES....10
V. SUPPLEMENTARY FIGURES....17

I. Supplementary Discussion

1. A Time Tree for Mosquitoes

Direct fossil evidence for the Culicidae dates to the Cretaceous, but fossils for mosquitoes are relatively rare [1]. The existence of abundant fossils placed in the sister lineage to mosquitoes, the Chaoboridae, in the early Jurassic[2] indicates that the direct lineage leading to mosquitoes is likely at least this old, despite lack of fossil evidence before the Cretaceous[1,3]. This ancient age for mosquitoes is in line with our estimates that they diverged from a common ancestor with Chaoboridae in the late Triassic 216 [95% HPD:188-249] million years ago.

The late Triassic origin and subsequent early Jurassic divergence to extant lineages of mosquitoes in our study (179 MYA, 95% HPD: 146-213) stands in stark contrast to the estimates of Misof et al.[4], which placed the origin of extant mosquito subfamilies in the early Cretaceous, with a median estimate of 75 MYA (CI: 7-152 MYA). Our estimates are also older than those of Tong et al.[5], which used MCMCtree, although these authors found a late Cretaceous divergence for extant mosquito subfamilies (125 MYA, 95% CI: 119-175). Both studies relied on the same

genomic dataset (primarily transcriptomes), and only two mosquitoes were sampled; this difference in sampling may explain the large discrepancy in age estimates between those studies and ours. A Jurassic origin of mosquitoes is consistent both with studies using only a handful of species, usually based on available *Aedes* and *Anopheles* genomes[6–8], and those using more extensive, multi-genera datasets[9,10]. However, our study is so far unique in its combination of taxonomic coverage of so many lineages, and robust inference from a genomic dataset, allowing insight into the evolution of particular mosquito lineages.

Our divergence time estimates, as well as previously paleontological evidence, and present-day distributions, strongly support a Gondwanan radiation for many mosquito lineages. In anophelines, for instance, a Gondawanan radiation explains both the endemic presence of *Chagasia* and many subgenera in *Anopheles* in the Neotropics. The endemism of *Bironella* to Australia and Papua-New Guinea and other species groups to Africa and India lend further support to our finding. Lastly, the ~100 MY old anopheline fossil, *Priscoculex burmanicus*, is from Burmese amber[3]. Burmese amber mines are located on the West Burma Block, which is thought to have drifted from Australia to Southeast Asia during the Cretaceous[11], thus reflecting Gondwanan origins.

Similarly in the Culicinae, many lineages have distributions remnant of Gondwanan radiations. For example, both the Aedini and the Culicini have endemic lineages found in the Neotropics and regions of east Asia. However, many of the earliest fossils in the Culicinae reveal this subfamily was likely globally widespread even during the Cretaceous, as the oldest fossil attributable to the Culicinae is *Paleoculicus minutus*, from Canadian Cretaceous amber[12]. By the Cenozoic, several fossils of Culicidae are attributable to extant genera of *Aedes*[13], *Culiseta*[14], *Culex*[13], and *Coquillettidia*[15,16], in line with our estimates of these extant genera evolving in the late Cretaceous or early Cenozoic.

2. The Evolution of Host Associations in Mosquitoes

Due to the global nature of our sampling and the high coverage of major extant lineages, our study allows novel insight into the relative and absolute divergences of lineages at a level previously not possible. The imprint of continental drift appears clear with our present sampling across the family, from New World anopheline lineages to aedines in *Psorophora*. But more than just apparent signals of biogeography, our analyses suggest divergences in line with major host lineage diversification and expansion. To investigate this possibility, we compiled a century of literature on mosquito host association and found striking patterns in the evolution of mosquitoes reflected in shifts in host association.

Most mosquito species exhibited a strong association to a single class of host, while others exhibited polyphagy across two or more host classes (Supplemetnary Figure 4 and Supplementary Figure 5). Most of our host association observations were of mammals, likely representing their dominance in terrestrial environments. However, we think that our observed host associations signify more than the relative abundance of vertebrate hosts where mosquitoes are found. Certainly, the relative abundance of hosts is important, and likely explains why there is some variability even in clear mammal specialists in feeding behaviors (e.g. *Anopheles gambiae* with 109 non-mammal observations out of 27,367), and how zoonotic vectors such as those in the genera *Culex*, *Mansonia*, and *Coquillettidia* can transmit diseases from avian reservoirs to mammals[17,18]. However, host association studies different genera with different

proportions of blood-meal sources from the same area (e.g. as in[19–21]), and perhaps more importantly, class-level mosquito-host associations had significant phylogenetic signal, indicating that genetically more similar mosquito species tended to have more similar host associations.

The strong host associations we demonstrated allowed us to interrogate ancestral feeding patterns in mosquitoes. We found strong evidence across a range of reconstructions for an amphibian-feeding ancestor of the Culicidae (Fig 2, Supplementary Figure 20-23). Amphibians were common terrestrial vertebrates during the Jurassic when mosquitoes evolved and could have provided ample sources of vertebrate blood in Jurassic wetlands. Interestingly, the nearest blood feeding relative of the Culicidae, the Corethrellidae, feed exclusively on amphibians. However, blood feeding is thought to have evolved independently in the ancestors of the Culicidae from entomophagy or plant-feeding[22], as the sister family to mosquitoes, Chaoboridae, do not blood-feed. Alternatively, as biting mouthparts may be ancestral in the Culicimorpha[23,24], it is possible that blood feeding was lost in the ancestors of the Chaoborids. Loss of biting behavior has occurred in some lineages in the Culicidae[25,26], but there is no morphological evidence of this in Chaoborids.

In the Culicinae, it is interesting that our models generally did not find strong support for a strong association with mammals or birds in most lineages until after the K-Pg, when these two major vertebrate clades radiated. Indeed, when comparing our ancestral state reconstruction to the changes in lineages through time for extant vertebrate clades (Fig 2 D), major shifts in host associations among mosquitoes do often occur when vertebrate lineages themselves become more common, perhaps indicating that the proliferation of these vertebrate host lineages enabled the same in mosquitoes.

In the Anophelinae, our reconstructions supported a mammal feeding ancestor for *Anopheles*, perhaps unsurprising given that all but two species in this genus had mammal bloodmeal records. This close association with mammals has enabled these mosquitoes to serve as major vectors for *Plasmodium* in mammals throughout their global range, causing severe disease in much of the tropics, and historically shaping human history in temperate regions, as well[17,27,28]. At first, this high degree of association to mammals prior to their major radiations may seem counterintuitive; however, based on our divergence time estimates, it seems quite likely that ancient *Anopheles* were found in west-central Gondwana during the Late Jurassic and Early Cretaceous, in what would become South America and Africa, where abundant ancient mammal fossils have been found[29]. As such, a host association with mammals could have evolved then.

A major limitation of our analyses on the evolution of host associations is our inability to account for potential feeding on non-avian dinosaurs; however, our estimates did suggest a strong possibility of reptile feeding among ancestral nodes of the Culicinae in the Cretaceous, which could reflect this. Moreover, although our analysis is the largest to date on host associations in mosquitoes, it still reflects only 422 of the approximately 3600 species of mosquitoes. However, given the strong phylogenetic association with host associations at the genus level, and our high degree of sampling at this level, we feel our analysis is robust to our present sample size. Moreover, our analysis demonstrates the possibilities that a phylogenomic backbone of mosquitoes present at better understanding the evolution of phenotypes critical to disease.

3. Systematics of the Culicidae

Phylogenomic datasets have resolved evolutionary relationships across many insect groups[4,30], and the same is true here in mosquitoes. Here, we present a brief discussion of major clades of mosquitoes, as well as touching on recent nomenclature controversies surrounding these groups. We demonstrate that a phylogenomic dataset may finally resolve long-standing controversies and provide a path forward for future systematic efforts and taxonomic revisions in mosquitoes.

The relationships inferred by our analyses confirmed the monophyly of the Culicidae and both subfamilies, as well as the monophyly of all tribes we sampled. While our findings with regards to monophyly of the family and subfamily largely correspond to existing knowledge of mosquitoes based on cladistics and natural history observations[31–34], cladistic analyses based on morphology have frequently questioned the monophyly of many tribes, particularly the Aedini[35,36], and have rarely provided strong or consistent evidence for the evolutionary relationships within subfamilies, among tribes, genera, or subgenera[36–38].

Our finding of an early divergence of *Chagasia* in the Anophelinae is unsurprising given the unique morphology[39] and chromosomal characteristics[40] of this mosquito and is consistent with prior hypotheses on the Anophelinae[9,37,41,42]. *Chagasia* has three pairs of autosomes and a pair of sex chromosomes (2n=8), the same chromosomal configuration as *Mochlonyx*[40,43,44], but different from all *Anopheles* (two pairs of autosomes and a sex chromosome). Moreover, despite sharing the same number of chromosomes as members of the Culicinae, Culicinae lack a sex chromosome, and chromosome content of the Culicinae and *Anopheles* suggests independent fusion events[43,44]. To our knowledge, there is no cytogenetic information on most early diverging Culicinae (most cytogenetic knowledge of Culicinae comes from *Culex* and *Aedes*), but despite this, it appears likely that *Chagasia* harbors an ancestral chromosomal configuration, consistent with its phylogenetic position.

We sampled six of eight *Anopheles* subgenera, and the two unsampled subgenera, *Baimaia* and *Christya*, have one and two species respectively. We found two major clades of *Anopheles*: the first is composed of subgenera *Stethomyia*, *Lophopodomyia*, *Kerteszia*, and *Nyssorhynchus*; the second contains *Anopheles* and *Cellia*. In our analysis, the first clade contains many American malarial vectors, particularly in South American countries, such as *Anopheles* (*Nyssorhynchus*) *darlingi* and *Anopheles* (*Nyssorhynchus) albimanus*. Morphological analyses did not usually reflect these two clades of *Anopheles* subgenera[37,45,46]. However, recent analyses based on mitochondrial genomes found similar relationships among New World subgenera to our phylogenomic data[10,42]. These subgenera were recently elevated to generic status by Foster et al.[42] after analyses suggested *Bironella* resolved within the genus *Anopheles* and sister to subgenera *Anopheles* + *Cellia* (but see[47]). Unfortunately, due to our inability to sample *Bironella*, we are unable to evaluate this decision with our molecular dataset, but other recent analyses of mitochondrial genomes suggest instead *Bironella* is sister to *Anopheles* sensu lato[10,48]. Interestingly, we find *Anopheles* (*Anopheles*) *stigmaticus* as diverging prior to all other *Anopheles*, thus rendering the subgenus *Anopheles* as paraphyletic. To our knowledge, this is the first genomic data generated from a member of the Stigmaticus Group, but morphological analyses also placed this group outside of *Anopheles* (*Anopheles*)[45].

Our phylogenomic analysis provided strong support to a backbone phylogeny of the Culicinae, which has remained contentious or unresolved, because of the ancient age of the group, and the lack of comprehensive molecular phylogenies to date. Morphology has presented conflicting resolution as to the relationships among tribes in the Culicinae[25], perhaps due to convergence in many morphological characters[49]. The cladistic analysis of 38 genera and 73 morphological characters conducted by Harbach and Kitching[36] did support the position of the Aedeomyiini and Uranotaeniini as early diverging genera in the Culicinae, consistent with our phylogenomic analyses. This is unsurprising in the case of Aedeomyiini, as this group has long been thought of as ancient. Belkin[31] remarked that "*Aedeomyia* is probably a very ancient primitive but highly specialized segregate...", referring to both their unique ornamented adults and specialized larval morphology with greatly swollen and elongate antennae. Interestingly, Chaoborids, as well as the Anophelinae, Aedeomyiini, and the Uranotaeniini—the first three branches of the culicids and the sister clade to mosquitoes—all have a completely or nearly completely membranous proctiger. Such a feature is also present in *Verrallina*, though, where it is certainly derived.

Relationships among Culicinae tribes have remained largely unresolved in the face of morphological analyses or the limited molecular analyses performed to date. Previous analyses of molecular data were restricted to either single marker datasets[50–52], multiple markers but limited taxon sampling[9,49], or genome-scale SNP or transcriptome datasets restricted to species groups in *Anopheles* or *Aedes* or otherwise limited in taxonomic breadth[53–57]. Perhaps the most comprehensive analysis of Culicinae phylogeny to date is from Reidenbach et al.[9], which used six genes and 80 morphological characters from three species in the Anophelinae and 23 in the Culicinae. Although this sampling of Culicinae reflected ten tribes, only two of those tribes, Sabethini and Aedini, had more than one species sampled, and their analysis had difficulty resolving relationships between most tribes, as many backbone nodes had low support values. Similarly, da Silva et al.[10] sampled mitochondrial genomes 37 species from 15 genera and 7 tribes but had difficulty resolving deep relationships in the Culicinae. However, as in our analyses, these authors found *Uranotaenia* and *Aedeomyia* as the earliest diverging lineages in the Culicinae. Furthermore, while their analysis did find different relationships among tribes in general, they also found a clade composed of Toxorhynchitini+Sabethini+Mansoniini, albeit with an extremely low branch support value leading to this clade (BPP=0.5). These three tribes are associated in our analyses, along with the Culisetini and Orthopodomyiini, both unsampled by da Silva. Our finding that the Toxorhynchitiini is sister to the Sabethini confirms speculation by Belkin in the 20th century that these two lineages were closely related[31].

Among sampled mosquito genera, all but *Aedes* and *Culex* were monophyletic. The classification of both genera has been contentious, likely because of their enormous size—*Aedes* is more than 900 species in a tribe that contains a third of all mosquito species (1266), while *Culex* contains 785 species in a tribe of 813 species(Mosquito Taxonomic Inventory).

The genus *Aedes*, as it is presently recognized, is defined by a larval and adult morphological characters[31,58–61]. Based on a series of cladistic analyses of morphological characters, nearly all species within the genus *Aedes* saw their generic epithet changed multiple times at the start of the twenty-first century[17,38,62]. There were varying degrees of acceptance to these taxonomic changes prior to the revision to an inclusive *Aedes* of more than 900 species in 2015[17,63–65]. This revision to an inclusive *Aedes* was not based on the monophyly of *Aedes* sensu lato but instead based on the utility of the nomenclature (e.g. phenotypic diagnosability), lack of stability of clades under

different analysis conditions, and partially, the lack of corroborating molecular evidence[17]. The lack of monophyly highlighted by cladistic analyses in *Aedes* had been long recognized, as first Dyar and then many others noted that there were two groups in the genus *Aedes*[31,61,62,64,66,67], and that these groups had strong affinity to other aedine genera, establishing that *Aedes* was not monophyletic[31,61,68]. Belkin noted the affinity of genera *Haemagogus* and *Opifex* to subgenera like *Finlaya* and *Ochlerotatus* in his Section A grouping of aedines, with affinity between *Heizmannia*, *Armigeres*, and others, and subgenera *Stegomyia* and *Aedes* reflected in his Section B[31]. These sections largely correspond to the two clades of *Aedes* recovered in our analysis and others[10,49,52].

Our analysis confirms that 1) *Aedes* in the traditional and present sense is not monophyletic, and 2) that this is the case for both groups of *Aedes* mosquitoes proposed by Reinert[62] as *Aedes* and *Ochlerotatus*. We also find several subgenera in *Aedes* (*Aedeomorphus*, *Ochlerotatus*, and *Finlaya*) are not monophyletic and thus caution against elevating all subgenera in *Aedes* to generic status as the only solution to resolving conflicts in *Aedes,* as was previously attempted in the early 21st century. Future analyses, ideally including more aedine genera and subgenera from *Aedes* may enable a robust classification of aedine mosquitoes based on molecular evidence that is consistent with morphological characters.

In the case of *Culex*, previous analyses based on either limited molecular or morphological datasets have highlighted that *Lutzia* and *Deinocerities* (with allied genus *Galindomyia*, which we did not sample) likely originated from within *Culex*[69–71], although other analyses recover these genera as outside *Culex*[72–74]. Owing to the unique ecology and morphology of these species, for instance *Lutzia*'s predatory larvae, they have been held as separate genera according to most mosquito taxonomy[34,75]. Navarro and Liria[69] noted the affinity of *Deinocerites* to *Melanoconion*, *Microculex*, and other subgenera, which we also observe here, and proposed the demotion of *Deinocerites* to subgeneric status. This action has largely been neglected by taxonomists, but our results certainly support this action. In our analysis, *Lutzia* resolves within *Culex* as sister to the subgenus *Culex* (Fig S1), while *Deinocerites* resolves as sister to *Micraedes*, in a clade with several other New World *Culex* subgenera including *Melanoconion* (Fig S 1). Molecular phylogenies have consistently found *Lutzia* and *Deinocerites* as resolving within the genus *Culex*[73,76,77], and where sampling was sufficient, typically with the same subgenera as in our analysis. Although we have relatively poor sampling of each genus (one and two species, respectively), given previous molecular and morphological analyses, it is clear that both *Lutzia* and *Deinocerites* resolve within *Culex*, and thus likely should be demoted to subgenera.

Our phylogenomic analyses set the stage for further work in mosquito systematics and taxonomy, which we hope can finally address long-standing disagreements in systematics and nomenclature which have plagued mosquito biology for the last century.

II. Supplementary Notes

1. Supplementary Note 1

Taxon Sampling, Quality Assessment, and Dataset Characteristics

Our dataset contained 263 mosquito species from six different continents, along with genomic data from 5 outgroup flies used for rooting or dating purposes. Our sampling included individuals from 24 of 41 mosquito genera and nine (of eleven) tribes in the Culicinae, sampling that reflects genera containing 3377 mosquito species, out of approximately 3600. We had at least two species per genus for 17 of the 24 genera sampled.

This phylogenomic sampling included 215 anchor hybrid samples with 42 genomes and 11 transcriptomes. Our sequencing efforts resulted in new genomic sequencing data for 224 mosquito species, including nine new low-coverage genomes assemblies and associated orthologs. Across all our samples, mean recovery of orthologs (Supplementary Figure 8) post contamination removal was highest for genomes (6307), and lowest for anchor hybrid enrichment samples (3584). However, due to the preponderance of anchor hybrid samples in our dataset, as well as the fact our anchor hybrid enrichment samples consistently recovered the 477 mosquito orthologs targeted by our probes (Supplementary Figure 8), there was no difference in the average number of loci in our primary amino acid alignment was similar across all three data types (S Table 2, F=2.294, Df=2, P=0.103). There was no significant difference in the number of orthologs we aligned between subfamilies (F=1.897, Df=2, P=0.152), and although there was a difference between tribes (F=2.299, Df=8, P=0.022), this was due to the large sample size differences between tribes (Fig S8).

Our primary amino acid alignment consisted of 709 orthologs found in 203 species or more and had a total alignment length of 523,035 amino acids. Per gene alignments were on average 737 amino acids long. Our automated distanced-based method of removing highly divergent/outlier gene sequences removed a total of 489 sequences from alignments (0.002% of total sequences). Removal of these outlier sequences had relatively little change in topology in most cases, but it helped resolve the place of Orthopodomyiini with the Culisetini; in our analysis of our untrimmed alignment, Orthopodomyiini resolved as sister Mansoniini, albeit with very low support. Additionally, our outlier removal method reduced terminal branch lengths of many taxa, resulting in a total reduction in tree size from 10.67 to 10.16 (a 5% reduction), including an increase in the relative length of internal branches.

2. Supplementary Note 2

Topological Congruency Across Analyses and the Influence of Saturation

The maximum likelihood phylogeny constructed from nucleotide position two had a congruent topology with those from our amino acid analyses, with the only difference being deep within clades (Supplementary Figure 2). We found strong support for the monophyly of both subfamilies and all tribes (systematics are discussed at length below). Similar topologies were recovered using various subsets or alternative datasets, although some differences did exist (Supplementary Figure 9-13). From our analysis of the 55 genomes and transcriptomes in our

dataset, we retrieved orthologs found in at least 75% of these species, resulting in a concatenated amino acid alignment of 2,459,406 amino acids from 5667 orthologs. We recovered similar relationships among tribes in this analysis as previously described maximum likelihood analyses, although this dataset lacked the same sampling and as such, we could not evaluate the relationships among tribes or genera as clearly. Moreover, we found a similar topology with coalescent-like analyses from ASTRAL based on genes trees from amino acid sequences (Supplementary Figure 11-13), although this analysis had low support values for backbone branches in the Culicinae. As we increased the number of tips required to include a gene tree to 80% (214 species) and 90% (241 species), some branch support values did increase, but others remained low. Our alternative analyses did occasionally recover topological differences, usually within clades, and often with low support. For instance, although our analysis of only orthologs targeted by probes recovered the same tribe and genus-level topology as our primary analyses (Fig S9), there were some differences within clades, such as *An. stigmaticus* as the earliest diverging branch in the genus *Anopheles*, albeit with only moderate SH-like aLRT branch support (89.9).

We found significant evidence of saturation at codon positions one and three, both of which exhibited a non-linear relationship between genetic distance and transition and transversion ratios (Supplementary Figure 6). The inclusion of nucleotide position one and/or three resulted in conflicting topologies to those of position two and amino acids. For instance, when codon positions one and two, the analysis supported a result wherein Toxorynchitinii was sister to Orthopodomyiini, which were together sister to Culiestini, albeit with extremely low support (61.2 for ultrafast bootstrap support, and 74 for SH-like aLRT). In contrast, the topology from amino acids and nucleotide two showed Toxorynchitnii was sister to Sabethini with high support (Fig 1, Supplementary Figure 1 and 2). We considered this difference to be a result of saturation.

As our primary analyses utilized amino acids and nucleotide position two to reconstruct evolutionary relationships that were hundreds of millions of years old and thus may be less informative at the tips of the phylogeny, we also reconstructed a phylogeny from all three nucleotide positions, partitioned by gene and codon position, in which the subfamily, tribe, and generic level relationships of mosquitoes were constrained to our primary topology. Species-level relationships remained largely unchanged, although some relationships did shift deep within genera, such as *Aedes (Hulecoeteomyia) japonicus*. This species was sister to the subgenera *Mucidus+Finlaya kochi + Gymnomtopea + Georgecraigius* in the amino acid topology, while this species resolves as sister to *Patmarksia+Dobrotworskyius* in a constrained first second, and third, position nucleotides topology.

3. Supplementary Note 3

Clock Selection, Divergence Time Estimation, and Convergence of Chains

We found strong support for the independent rates relaxed clock model (i.e., posterior model probability > 0.92; Supplementary Table 3), so subsequent divergence time estimation used this clock model. Our analyses in MCMCTree ran for a combined total of 23,712,000 generations across five chains (Fig S14), with each chain running for approximately 384 hours for a total computation time of approximately 1,920 hours. The estimated sample size of most node and parameter estimates exceeded 500 and only 12 were below 200, all of which were at least 190.

4. Supplementary Note 4

Database of Host Associations

We recorded 293,308 mosquito-host associations representing data from 435 species in 25 genera. We recovered blood-host associations from all subfamilies and all tribes of biting mosquitoes. Virtually all observations were recorded at the species level of the mosquito, but unfortunately, some were listed only as "sp" and were discarded from additional analyses (12 unidentified species with 352 total observations), resulting in 422 mosquitoes for which we had host-associations. Among these 422, all but two fed predominantly on one of the four classes of terrestrial vertebrate hosts: Aves, Mammalia, Reptilia, or Amphibia. However, two species were exceptions: *Uranotaenia sapphirina*, which had 83 blood-meal observations of which 80 annelids[21] and *Aedes baisai*, which had 283 observations of which 280 were fish[78]. While we did not exclude these species, we did not include host association categories for fish or annelids in our dataset. These 422 species also contained representatives from two tribes of mosquitoes we were unable to sample with molecular data: Ficalbiini (7 species with 66 observations) and Hodesgiini (1 species with 1 observation).

Most species had multiple host-association observations (median = 46 observations, Supplementary Figure 15). Due to the fact that species of medical importance typically had far higher raw observation counts (e.g. *Anopheles gambiae* had 27,376 and *Culex tritaeniorhynchus* had 29,597), we used a proportion of observations for each class in our analyses. In general, mosquitoes tended to have a strong association with a single class of host (mean max host association = 0.90, Supplementary Figures 4 and 5), but there were exceptions, particularly in the genus *Culex*.

5. Supplementary Note 5

Phylogenetic Comparative Methods

We found significant phylogenetic signal across host associations in mosquitoes, as measured by Blomberg's K and a phylogenetic correlogram, both with and without Anophelinae. When we separately considered each host class, Amphibia showed greatest the effect size of phylogenetic signal (Supplementary Figures 16-20). We found significant and strong positive correlation at lower genetic differences—evidence of similarity in host association at low genetic distances between any two mosquitoes, and a significant negative correlation at higher genetic differences—evidence of dissimilarity in host association between mosquitoes more distant related.

For stochastic character mapping, we evaluated different models of character evolution, and found that the model where all transition rates differed (ARD) was the best model based on AICc weights. Using the ARD model across a range of reconstructions, we found the highest posterior probability was for an amphibian feeding ancestor of the Culicidae and Culicinae (Fig 2). This was true whether we included tribes we did not sample with our molecular backbone (Ficalbiini and Hodgesiini) or not (Fig 2 and Supplementary Figure 21), and when we used a flat prior for mosquitoes where we lacked host association data (Supplementary Figure 22), as well as when we estimated stochastic character maps across a posterior of TACT reconstructions (Supplementary Figure 23). While the degree of support for an amphibian feeding ancestor for

the Culicidae and Culicinae did vary between these reconstructions, it was always the highest posterior probability. Moreover, outside the Anophelinae, most reconstructions found that mammal and bird feeding greatly increased as the supported ancestral state for clades starting at about 65 MYA.

III. Supplementary References

1. Borkent, A. & Grimaldi, D. A. The Earliest Fossil Mosquito (Diptera: Culicidae), in Mid-Cretaceous Burmese Amber. *Annals of the Entomological Society of America* **97**, 882–888 (2004).
2. Borkent, A. World catalog of extant and fossil Chaoboridae (Diptera). *Zootaxa* **3796**, 469–493 (2014).
3. Poinar, G., Zavortink, T. J. & Brown, A. *Priscoculex burmanicus* n. gen. et sp. (Diptera: Culicidae: Anophelinae) from mid-Cretaceous Myanmar amber. *Historical Biology* **32**, 1157–1162 (2020).
4. Misof, B. *et al.* Phylogenomics resolves the timing and pattern of insect evolution. *Science* **346**, 763–767 (2014).
5. Tong, K. J., Duchêne, S., Ho, S. Y. W. & Lo, N. Comment on "Phylogenomics resolves the timing and pattern of insect evolution". *Science* **349**, 487–487 (2015).
6. Krzywinski, J., Grushko, O. G. & Besansky, N. J. Analysis of the complete mitochondrial DNA from *Anopheles funestus*: An improved dipteran mitochondrial genome annotation and a temporal dimension of mosquito evolution. *Molecular Phylogenetics and Evolution* **39**, 417–423 (2006).
7. Moreno, M. *et al.* Complete mtDNA genomes of *Anopheles darlingi* and an approach to anopheline divergence time. *Malar J* **9**, 1–13 (2010).
8. Chen, X.-G. *et al.* Genome sequence of the Asian Tiger mosquito, *Aedes albopictus* , reveals insights into its biology, genetics, and evolution. *Proc. Natl. Acad. Sci. U.S.A.* **112**, (2015).
9. Reidenbach, K. R. *et al.* Phylogenetic analysis and temporal diversification of mosquitoes (Diptera: Culicidae) based on nuclear genes and morphology. *BMC Evol Biol* **9**, 298 (2009).

10. da Silva, A. F. *et al.* Culicidae evolutionary history focusing on the Culicinae subfamily based on mitochondrial phylogenomics. *Sci Rep* **10**, 18823 (2020).
11. Poinar, G. Burmese amber: evidence of Gondwanan origin and Cretaceous dispersion. *Historical Biology* **31**, 1304–1309 (2019).
12. Poinar, G. O., Zavortinik, T. J., Pike, T. & Johnston, P. A. *Paleoculicis minututs* (Diptera: Culicidae) n. Gen., n. Sp., from Cretaceous Canadian amber, with a summary of described fossil mosquitoes. *Acta Geologica Hispanica* 119–130 (2000).
13. Szadziewski, R. & Giłka, W. A new fossil mosquito, with notes on the morphology and taxonomy of other species reported from Eocene Baltic amber (Diptera: Culicidae). *Pol. J. Entomol. Pol. Pismo Entomol.* **80**, 765–777 (2011).
14. Harbach, R. E. & Greenwalt, D. Two Eocene species of *Culiseta* (Diptera: Culicidae) from the Kishenehn Formation in Montana. *Zootaxa* **3530**, 25–34 (2012).
15. Krzemiński, W. *et al.* True flies (Insecta: Diptera) from the late Eocene insect limestone (Bembridge Marls) of the Isle of Wight, England, UK. *Earth and Environmental Science Transactions of The Royal Society of Edinburgh* **110**, 495–554 (2019).
16. Szadziewski, R., Sontag, E. & Szwedo, J. Mosquitoes of the extant avian malaria vector *Coquillettidia* Dyar, 1905 from Eocene Baltic amber (Diptera: Culicidae). *Palaeoentomology* **2**, 650–656 (2019).
17. Wilkerson, R. C. *et al.* Making Mosquito Taxonomy Useful: A Stable Classification of Tribe Aedini that Balances Utility with Current Knowledge of Evolutionary Relationships. *PLOS ONE* **10**, e0133602 (2015).
18. Foster, W. A. & Walker, E. D. Chapter 15 - Mosquitoes (Culicidae). in *Medical and Veterinary Entomology (Third Edition)* (eds. Mullen, G. R. & Durden, L. A.) 261–325 (Academic Press, 2019). doi:10.1016/B978-0-12-814043-7.00015-7.

19. Estep, L. K. *et al.* A Multi-Year Study of Mosquito Feeding Patterns on Avian Hosts in a Southeastern Focus of Eastern Equine Encephalitis Virus. *The American Journal of Tropical Medicine and Hygiene* **84**, 718–726 (2011).

20. Shepard, J. J., Andreadis, T. G., Thomas, M. C. & Molaei, G. Host associations of mosquitoes at eastern equine encephalitis virus foci in Connecticut, USA. *Parasites Vectors* **9**, 1–10 (2016).

21. Reeves, L. E. *et al.* Identification of *Uranotaenia sapphirina* as a specialist of annelids broadens known mosquito host use patterns. *Commun Biol* **1**, 1–8 (2018).

22. Peach, D. A. H. & Gries, G. Mosquito phytophagy – sources exploited, ecological function, and evolutionary transition to haematophagy. *Entomologia Experimentalis et Applicata* **168**, 120–136 (2020).

23. Borkent, A. The Pupae of the Biting Midges of the World (Diptera: Ceratopogonidae), With a Generic Key and Analysis of the Phylogenetic Relationships Between Genera. *Zootaxa* **3879**, 1–327 (2014).

24. Narayanan Kutty, S., Wong, W. H., Meusemann, K., Meier, R. & Cranston, P. S. A phylogenomic analysis of Culicomorpha (Diptera) resolves the relationships among the eight constituent families. *Systematic Entomology* **43**, 434–446 (2018).

25. Armbruster, P. A. Molecular pathways to nonbiting mosquitoes. *Proceedings of the National Academy of Sciences* **115**, 836–838 (2018).

26. Bradshaw, W. E. *et al.* Evolutionary transition from blood feeding to obligate nonbiting in a mosquito. *PNAS* **115**, 1009–1014 (2018).

27. Piel, F. B. *et al.* Global distribution of the sickle cell gene and geographical confirmation of the malaria hypothesis. *Nat Commun* **1**, 1–7 (2010).

28. Elguero, E. *et al.* Malaria continues to select for sickle cell trait in Central Africa. *Proc. Natl. Acad. Sci. U.S.A.* **112**, 7051–7054 (2015).

29. Pascual, R. & Ortiz-Jaureguizar, E. The Gondwanan and South American Episodes: Two Major and Unrelated Moments in the History of the South American Mammals. *J Mammal Evol* **14**, 75–137 (2007).

30. Peters, R. S. *et al.* Evolutionary History of the Hymenoptera. *Current Biology* **27**, 1013–1018 (2017).

31. Belkin, J. N. The Mosquitoes of the South Pacific (Diptera, Culicidae), Vol. 2. *The Mosquitoes of the South Pacific (Diptera, Culicidae), Vol. 2* (1962).

32. Wood, D. M. & Borkent, A. Phylogeny and classification of the Nematocera. in *Manual of Nearctic Diptera* (ed. McAlpine, J. F.) 1333–1370 (Research Branch, Agriculture Canada, 1989).

33. Harbach, R. E. The Culicidae (Diptera): a review of taxonomy, classification and phylogeny*. *Zootaxa* **1668**, 591–638 (2007).

34. Wilkerson, R. C., Linton, Y.-M. & Strickman, D. *Mosquitoes of the World*. (JHU Press, 2021).

35. Ross, H. H. Conflict with Culex. *Mosquito News* **11**, 128–132 (1951).

36. Harbach, R. E. & Kitching, I. J. Phylogeny and classification of the Culicidae (Diptera). *Systematic Entomology* **23**, 327–370 (1998).

37. Sallum, M. A. M., Schultz, T. R. & Wilkerson, R. C. Phylogeny of Anophelinae (Diptera Culicidae) Based on Morphological Characters. *Annals of the Entomological Society of America* **93**, 745–775 (2000).

38. Reinert, J. F., Harbach, R. E. & Kitching, I. J. Phylogeny and classification of tribe Aedini (Diptera: Culicidae). *Zoological Journal of the Linnean Society* **157**, 700–794 (2009).

39. Harbach, R. E. & Howard, T. M. Review of the genus *Chagasia* (Diptera: Culicidae: Anophelinae). *Zootaxa* **2210**, 1–25 (2009).

40. Kreutzer, R. D. A mosquito with eight chromosomes: *Chagasia bathana* Dyar. *Mosquito News* **38**, (1978).

41. Sallum, M. a. M. *et al.* Phylogeny of Anophelinae (Diptera: Culicidae) based on nuclear ribosomal and mitochondrial DNA sequences. *Systematic Entomology* **27**, 361–382 (2002).

42. Foster, P. G. *et al.* Phylogeny of Anophelinae using mitochondrial protein coding genes. *Royal Society Open Science* **4**, 170758 (2017).

43. Rao, P. N. & Rai, K. S. Comparative Karyotypes and Chromosomal Evolution in Some Genera of Nematocerous (Diptera: Nematocera) Families1. *Annals of the Entomological Society of America* **80**, 321–332 (1987).

44. Rai, K. S. & Black, W. C. Mosquito genomes: structure, organization, and evolution. *Adv Genet* **41**, 1–33 (1999).

45. Harbach, R. E. & Kitching, I. J. Reconsideration of anopheline mosquito phylogeny (Diptera: Culicidae: Anophelinae) based on morphological data. *Systematics and Biodiversity* **3**, 345–374 (2005).

46. Harbach, R. E. & Kitching, I. J. The phylogeny of Anophelinae revisited: inferences about the origin and classification of *Anopheles* (Diptera: Culicidae). *Zoologica Scripta* **45**, 34–47 (2016).

47. Harbach, R. E. An *Anopheles* by Any Other Name …? *Journal of Medical Entomology* **55**, 1069–1070 (2018).

48. Lorenz, C., Alves, J. M. P., Foster, P. G., Suesdek, L. & Sallum, M. A. M. Phylogeny and temporal diversification of mosquitoes (Diptera: Culicidae) with an emphasis on the Neotropical fauna. *Systematic Entomology* **46**, 798–811 (2021).

49. Soghigian, J., Andreadis, T. G. & Livdahl, T. P. From ground pools to treeholes: convergent evolution of habitat and phenotype in *Aedes* mosquitoes. *BMC Evolutionary Biology* **17**, 262 (2017).

50. Besansky, N. J. & Fahey, G. T. Utility of the white gene in estimating phylogenetic relationships among mosquitoes (Diptera: Culicidae). *Molecular Biology and Evolution* **14**, 442–454 (1997).

51. Isoe, J. Comparative analysis of the vitellogenin genes of the Culicidae. (The University of Arizona, 2000).

52. Shepard, J. J., Andreadis, T. G. & Vossbrinck, C. R. Molecular Phylogeny and Evolutionary Relationships Among Mosquitoes (Diptera: Culicidae) from the Northeastern United States Based on Small Subunit Ribosomal DNA (18S rDNA) Sequences. *JOURNAL OF MEDICAL ENTOMOLOGY* **43**, 12 (2006).

53. Zhou, X., Rinker, D. C., Pitts, R. J., Rokas, A. & Zwiebel, L. J. Divergent and Conserved Elements Comprise the Chemoreceptive Repertoire of the Nonblood-Feeding Mosquito Toxorhynchites amboinensis. *Genome Biology and Evolution* **6**, 2883–2896 (2014).

54. Fontaine, M. C. *et al.* Extensive introgression in a malaria vector species complex revealed by phylogenomics. *Science* **347**, 1258524 (2015).

55. Neafsey, D. E. *et al.* Highly evolvable malaria vectors: The genomes of 16 Anopheles mosquitoes. *Science* **347**, 1258522 (2015).

56. Thawornwattana, Y., Dalquen, D. & Yang, Z. Coalescent Analysis of Phylogenomic Data Confidently Resolves the Species Relationships in the *Anopheles gambiae* Species Complex. *Molecular Biology and Evolution* **35**, 2512–2527 (2018).

57. Soghigian, J. *et al.* Genetic evidence for the origin of *Aedes aegypti*, the yellow fever mosquito, in the southwestern Indian Ocean. *Molecular Ecology* **29**, 3593–3606 (2020).

58. Dyar, H. G. & Knab, F. The Larvæ of Culicidæ Classified as Independent Organisms. *J. N. Y. Entomol. Soc.* **14**, 169–230, 242 (1906).

59. Edwards, F. W. Notes On Culicidae, with Descriptions of New Species. *Bull. Entomol. Res.* **7**, 201–229 (1917).

60. Howard, L. O., Knab, F. & Dyar, H. G. *The Mosquitoes of North and Central America and the West Indies*. (Carnegie Institution of Washington, 1917).

61. Dyar, H. G. *The Mosquitoes of the Americas.* (Carnegie Institution of Washington, 1928).

62. Reinert, J. F. New classification for the composite genus *Aedes* (Diptera: Culicidae: Aedini), elevation of subgenus *Ochlerotatus* to generic rank, reclassification of the other subgenera. and notes on certain subgenera and species. *Journal of the American Mosquito Control Association* **16**, 175–188 (2000).

63. Black, W. C. Learning to use *Ochlerotatus* is just the beginning. *J Am Mosq Control Assoc* **20**, 215–216 (2004).

64. Savage, H. M. & Strickman, D. The genus and subgenus categories within Culicidae and placement of *Ochlerotatus* as a subgenus of *Aedes*. *J Am Mosq Control Assoc* **20**, 208–214 (2004).

65. Savage, H. M. Classification of Mosquitoes in Tribe Aedini (Diptera: Culicidae): Paraphylyphobia, and Classification Versus Cladistic Analysis. *Journal of Medical Entomology* **42**, 923–927 (2005).

66. Dyar, H. G. The Male Genitalia of Aëdes as Indicative of Natural Affinities (Diptera, Culicidae). *Insecutor Inscitiae Menstruus* **6**, (1918).

67. Edwards, F. W. A Revision of the Mosquitos of the Palaearctic Region. *Bulletin of Entomological Research* **12**, 263–351 (1921).

68. Edwards, F. W. Genera insectorum, Diptera. Fam. Culicidae. *Fascicle* **194**, 258 (1932).

69. Navarro, J.-C. & Liria, J. Phylogenetic relationships among eighteen neotropical Culicini species. *Journal of the American Mosquito Control Association* **16**, 75–85 (2000).

70. St John, O. Phylogeny of the genus Culex (Diptera: Culicidae). (Department of Life Sciences, Imperial College London and the Natural History …, 2007).

71. Deus, S. Phylogeny of selected species of subgenus *Culex* mosquitoes (Diptera: Culicidae) from the U.S., Puerto Rico, and Guatemala, based on nucleotide sequences from three genes. (Colorado State University, 2009).

72. Harbach, R. E., Kitching, I. J., Culverwell, C. L., Dubois, J. & Linton, Y.-M. Phylogeny of mosquitoes of tribe Culicini (Diptera: Culicidae) based on morphological diversity. *Zoologica Scripta* **41**, 499–514 (2012).

73. Laurito, M. & Almiron, W. R. Phylogenetic relationships of *Culex* (*Culex*) species (Diptera, Culicidae) from Argentina based on morphological characters. (2013) doi:10.11646/zootaxa.3652.1.4.

74. Kitching, I. J., Culverwell, C. L. & Harbach, R. E. The phylogenetic conundrum of *Lutzia* (Diptera: Culicidae: Culicini): a cautionary account of conflict and support. *Insect Systematics & Evolution* **46**, 269–290 (2015).

75. Harbach, R. E. *Culicipedia: Species-group, genus-group and family-group names in Culicidae (Diptera)*. (CABI, 2018).

76. Demari-Silva, B., Vesgueiro, F. T., Sallum, M. A. M. & Marrelli, M. T. Taxonomic and Phylogenetic Relationships Between Species of the Genus *Culex* (Diptera: Culicidae) From Brazil Inferred From the Cytochrome c Oxidase I Mitochondrial Gene. *Journal of Medical Entomology* **48**, 272–279 (2011).

77. Vesgueiro, F. T., Demari-Silva, B., Malafronte, R. dos S., Sallum, M. A. M. & Marrelli, M. T. Intragenomic variation in the second internal transcribed spacer of the ribosomal DNA of

species of the genera *Culex* and *Lutzia* (Diptera: Culicidae). *Mem. Inst. Oswaldo Cruz* **106**, 01–08 (2011).

78. Miyake, T. *et al.* Bloodmeal host identification with inferences to feeding habits of a fish-fed mosquito, *Aedes baisasi*. *Sci Rep* **9**, 4002 (2019).

79. Wiegmann, B. M. *et al.* Episodic radiations in the fly tree of life. *Proceedings of the National Academy of Sciences* **108**, 5690–5695 (2011).

80. Szadziewski, R. & Szadziewski, M. M. *Culex erikae* sp. n. (Diptera, Culicidae) from the Baltic amber. *Polskie Pismo Entomologiczne* **55**, 513–518 (1985).

IV. SUPPLEMENTARY TABLES

Supplementary Table 1: Calibrations Used in Divergence Time Estimates

Species	Reference	Age Used	MCMCtree Notation	Additional Details
N/A - Root	Wiegmann et al.[79]	<250 MYA	<250	Noted as Calibration 1 on Fig S3. We placed a maximum age constraint on the root node of 250 MYA based on Wiegmann et al. 2011
Chaoboridae	Borkent and Grimaldi[1]	187 MYA	L(187,0.1,1,0.025)	Noted as Calibration 2 on Fig S3. The Chaoboridae have a rich fossil history, with more fossil occurrences than extant species, and are known from fossil deposits as old as the Early Jurassic (>183 MYA, see Kalugina and Kovalev 1 985, Rohdendorf 1962, Borkent 1993, and Borkent 2014). Borkent and Grimaldi 2004 considered 187 MYA to be an approximate age for the Chaoboridae. Due to our minimal sampling of the Chaoboridae, we considered this calibration a stem calibration for chaoborids, and placed it on the last common ancestor between Culicidae and Chaoboridae.

Priscoculex burmanicus Poinar, Zavortink, & Brown, 2019	Poinar et al.[11]	98.17 MYA	L(98.17,0.1,1,0.025)	Noted as Calibration 3 on Fig S3. *Priscoculex burmanicus* is known from mid-Cretaceous amber. A predominance of anopheline features allowed the assignment of this species to the Anophelinae, but has some features that distinguish it from present-day anophelines. As such, we considered this a stem anopheline and placed a calibration on the last common ancestor of the extant subfamilies based on the age of this fossil.
Culiseta lemniscata Harbach & Greenwalt, 2012	Harbach and Greenwalt[14]	46.2 MYA	L(46.2,0.1,1,0.025)	Noted as Calibration 4 on Fig S3. *Culiseta lemniscata* is known from a compression fossil in a shale deposit of the Kishenehn Basic, dated to the Eocene. The species can not be placed within an extant subgenus, but numerous subcostal setae indicate it likely has an affinity to subgenus *Culicella*, and apparently keys to the subgenus *Culicella*, though it possesses some characteristics not found in other members of this subgenus and instead belonging to *Climacura*. We thus placed this fossil at the node above the common ancestor of *Culicella* and *Climacura*, which was the common ancestor of all sampled *Culiseta*.

Aedes serafini Szadziewski 1998	Szadziewski and Gilka[13]	33.9 MYA	L(33.9,0.1,1,0.025)	Noted as Calibration 5 on Fig S3. *Aedes serafini* is described as having a gonocoxite with a distinct apical inner lobe, similar to other members of the subgenus *Ochlerotatus*. The authors specifically mention similarity to *Aedes (Ochlerotatus) excrucians*, a holarctic species. This is a Baltic amber fossil dated to the Eocene. We placed this fossil at the last common ancestor of the subgenus *Ochlerotatus*.
Coquillettidia gedanica Szadziewski, Sontag & Szwedo, 2019	Szadziewski, Sontag & Szwedo[16]	33.9 MYA	L(33.9,0.1,1,0.025)	Noted as Calibration 6 on Fig S3. *Coquillettidia gedanica* was found in Baltic amber and has been placed in the extant genus Coquilletidia. We placed this fossil as a calibration on the last common ancestor of the Mansoniini, as these two genera are closely related and can be difficult to distinguish.
Culex (Culex) erikae Szadziewski & Szadziewska, 1985	Szadziewski & Szadziewska[80]	33.9 MYA	L(33.9,0.1,1,0.025)	Noted as Calibration 7 on Fig S3. *Culex erikae* is known from Baltic amber. This species has been found in multiple amber occlusions, is morphologically quite similar to *Culex (Culex) pipiens* adults, and has been placed in the subgenus *Culex*. We placed this fossil on the common ancestor of *Culex (Culex)* and *Culex (Culicimoyia)*, the subgenus sister to *Culex (Culex)* in our analysis.

Supplementary Table 2: Comparing Ortholog Retrieval Across Genomic Data Source, Mosquito Subfamily, and Tribe.

	**Anchor Hybrid Enrichment**			**Gene Sets from Genomes**			**Transcriptomes**			**Totals**		
	**N**	**In Alignment**	**Probe Targeted**	**N**	**In Alignment**	**Probe Targeted**	**N**	**In Alignment**	**Probe Targeted**	**N**	**In Alignment**	**Probe Targeted**
**Outgroups**												
				4	0.75	0.75	1	0.91	0.92	5	0.78	0.78
**Anophelinae**												
No Tribes	22	0.90	0.94	26	0.93	0.90	3	0.71	0.71	51	0.90	0.91
**Culicinae**												
Aedeomyiini	1	0.90	0.91	1	0.84	0.76				2	0.87	0.83
Aedini	117	0.91	0.95	6	0.90	0.84	3	0.82	0.77	126	0.91	0.94
Culicini	44	0.88	0.94	4	0.85	0.80	1	0.98	0.98	49	0.88	0.93

Culisetini	8	0.78	0.93	1	0.94	0.87				9	0.80	0.92
Mansoniini	7	0.75	0.82							7	0.75	0.82
Orthopodomyiini	2	0.83	0.93							2	0.83	0.93
Sabethini	8	0.89	0.91				2	0.78	0.79	10	0.87	0.89
Toxorhynchitini	3	0.89	0.92				1	0.81	0.82	4	0.87	0.89
Uranotaeniini	3	0.92	0.94							3	0.92	0.94

In Alignment refers to the proportion of alignment orthologs found, on average, in that taxonomic grouping.
Probe Targeted refers to the proportion of probe-targeted orthologs found, on average, in that taxonomic grouping.

Supplementary Table 3: Clock Selection in MCMCTree

Model	Bayes Factor	log(Bayes Factor)	Pr(M|D)
ILN	1	0	0.92
GBM	0.08	-2	0.7
CLK	0	-7153	0

Supplementary Table 4: Bloodmeals Grouped into Reptiles, By Mosquito Genus					
	Clades grouped into Reptile in our Analyses				
Mosquito Genus	Crocodilia	Unreported	Squamata	Testudines	Total
Aedeomyia		1			1
Aedes		9	5	13	27
Anopheles		1	2	1	4
Coquillettidia		3		2	5
Culex	9	32	55	11	107
Culiseta		4	6		10
Deinocerites			7		7
Mansonia		1	1		2
Psorophora				2	2
Uranotaenia		3	5	1	9
	9	54	81	30	174

V. SUPPLEMENTARY FIGURES

Supplementary Figure 1

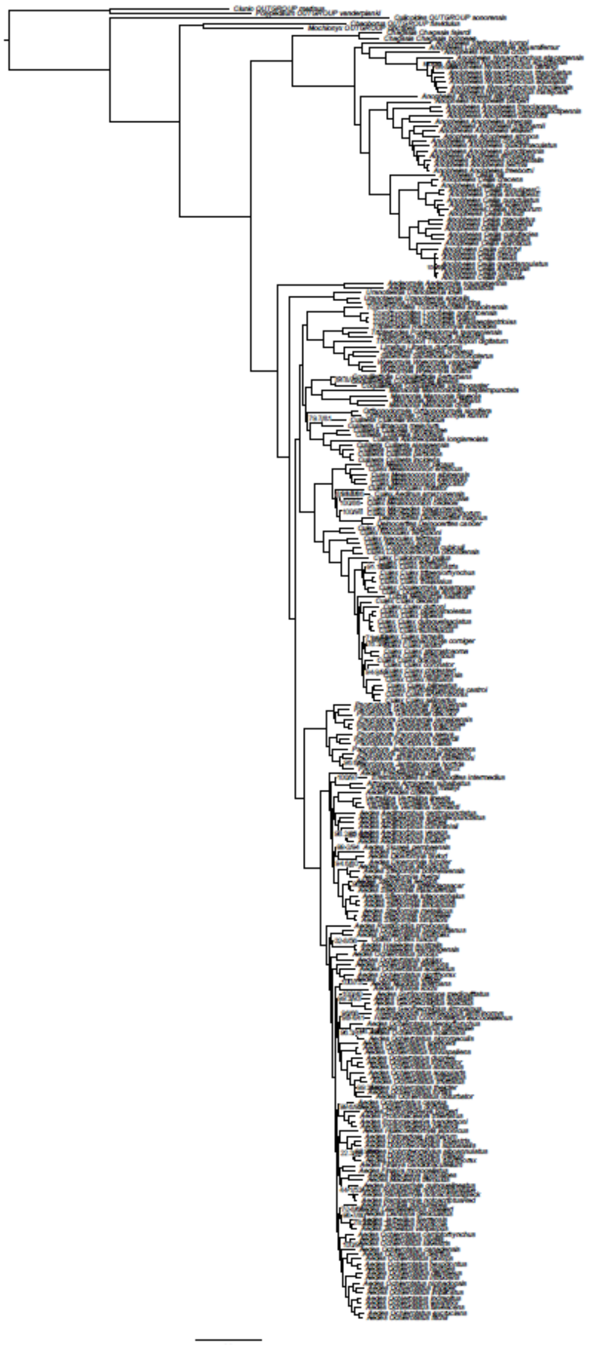

The maximum likelihood topology from IQ-Tree, based on partitioned amino acid alignment. Here, the model for each partition was chosen based on individual analyses of gene trees. Support values are SH-like aLRT and Ultrafast Bootstrap values. Values above 95/95 have been hidden. The scale is in amino acid substitutions per site.

Supplementary Figure 2

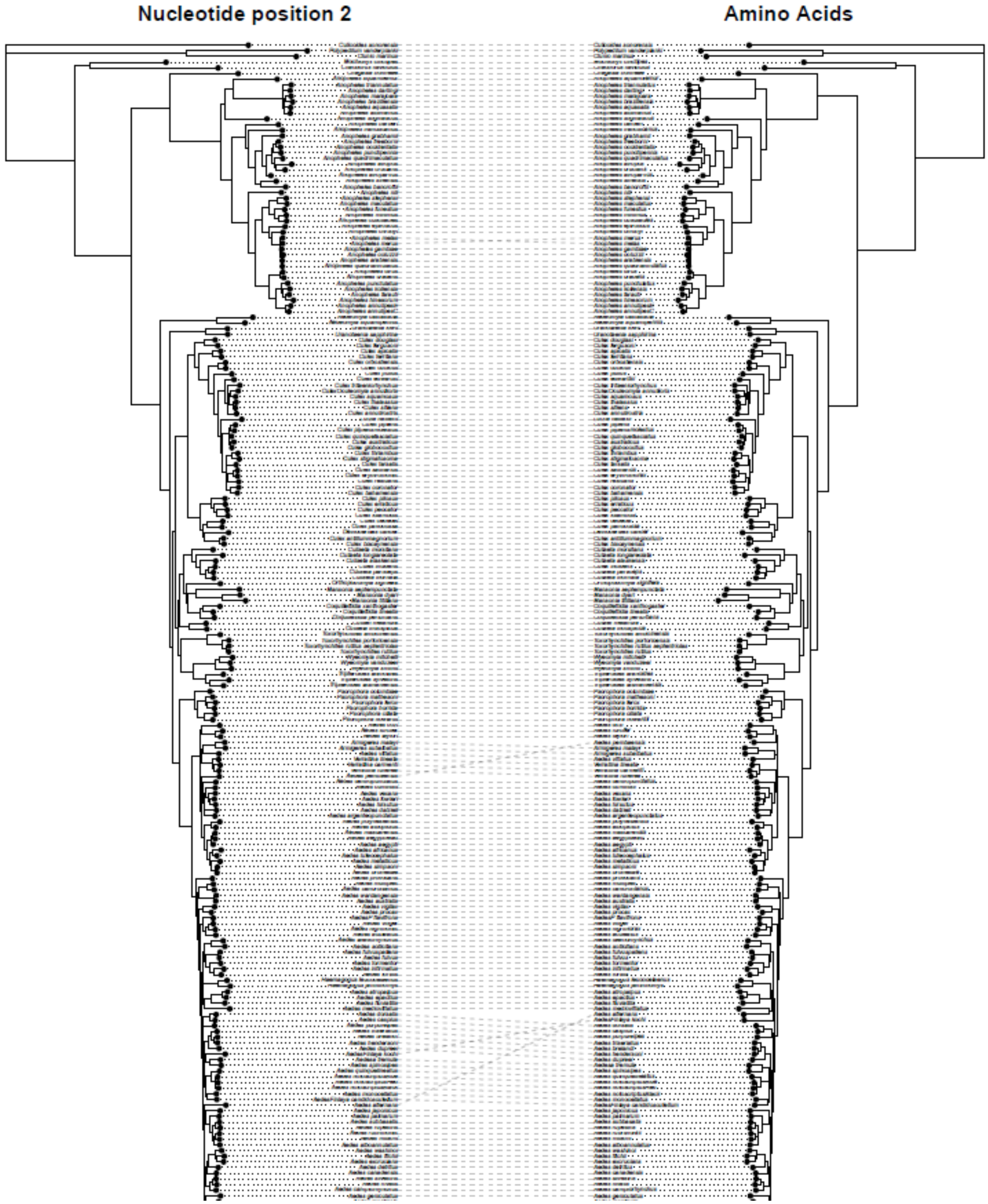

A comparison between the maximum likelihood topologies recovered by IQ-Tree based on partitioned analyses of nucleotide position two and amino acids. Dotted lines connect the same tips. The only differences are deep within clades.

Supplementary Figure 3

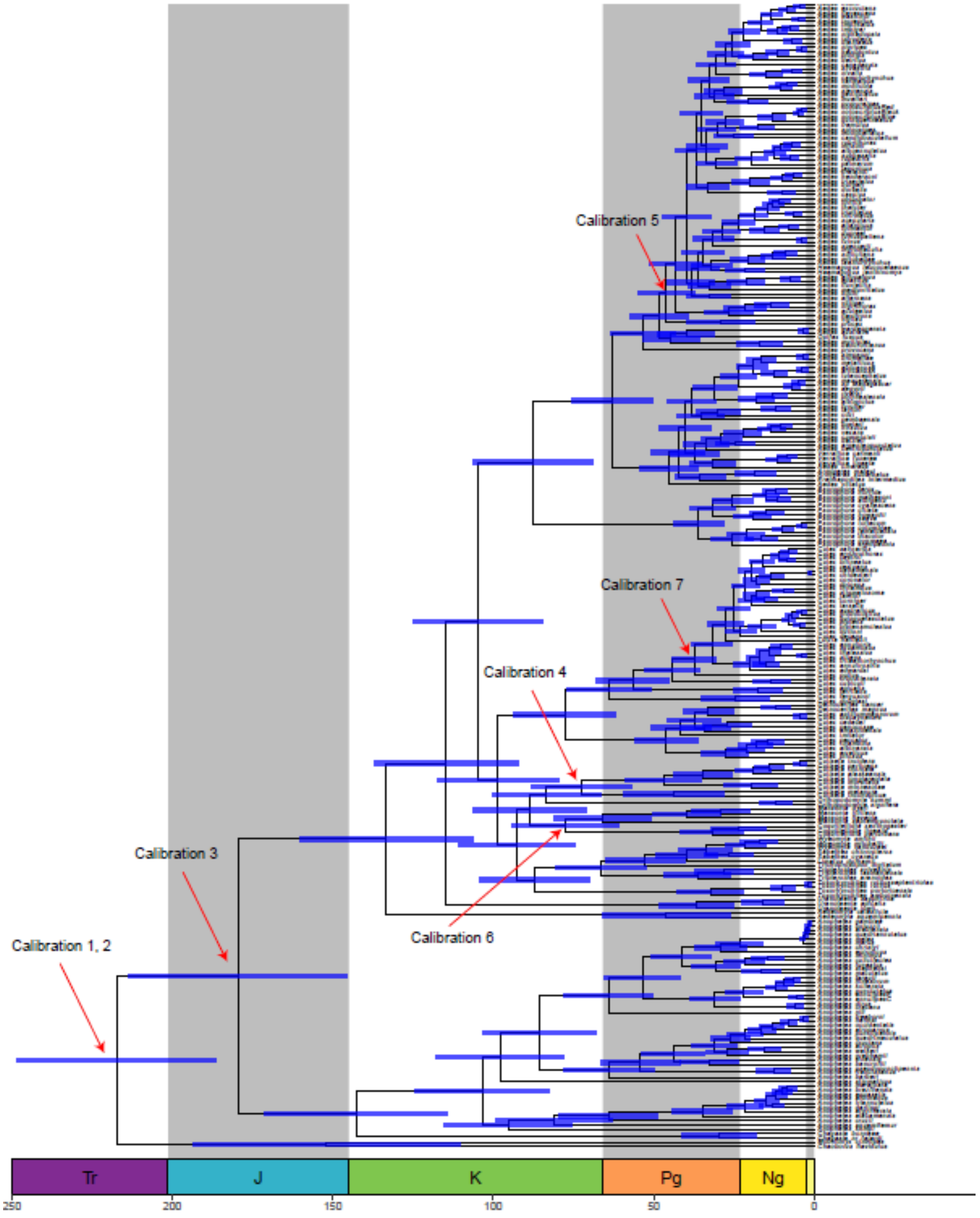

The divergence time analysis, from Figure 1, at a higher resolution and with species names visible on tips and calibration points shown with red arrows. Calibration details are given in Supplementary Table 1.

Supplementary Figure 4

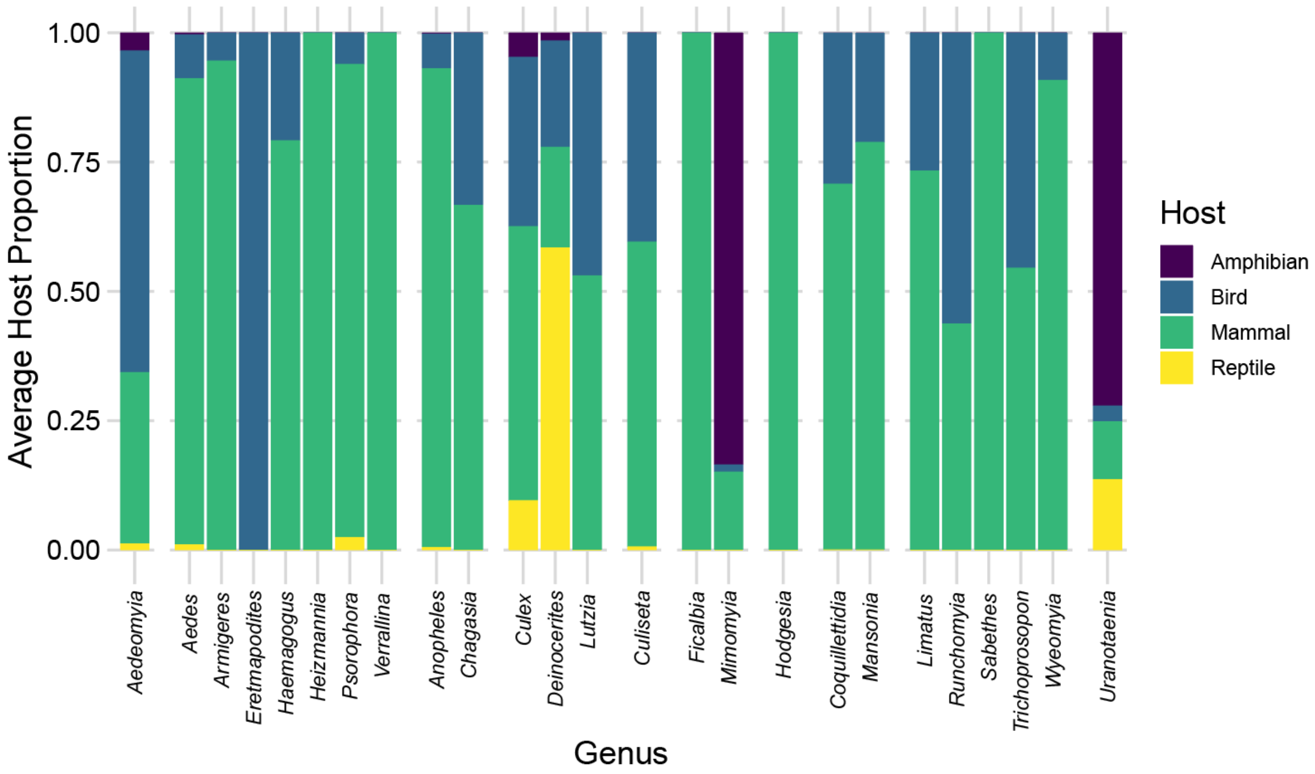

The average source of mosquito blood meals, by class, for each mosquito genus. Here, the average was calculated across all species within a given genus, regardless of number of observations.

Supplementary Figure 5

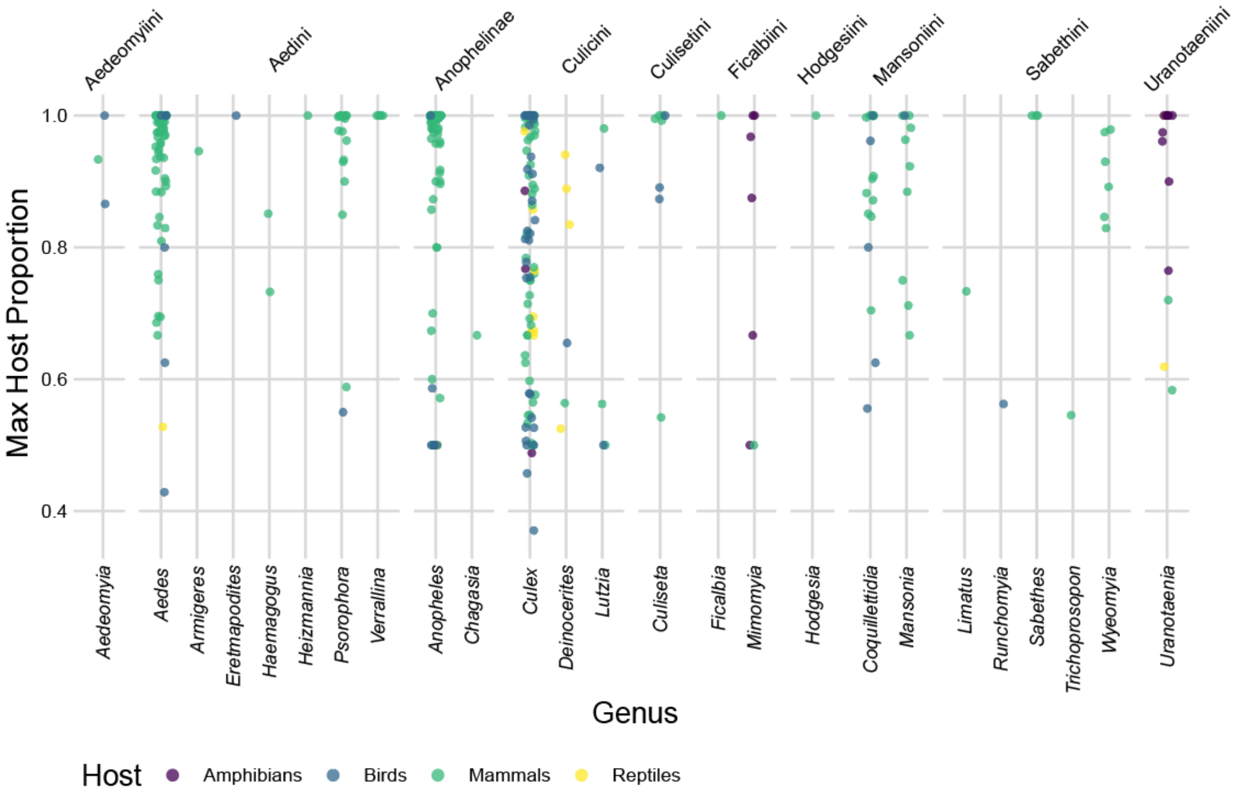

The maximum host proportion for each genus of mosquito, grouped by tribe or by subfamily for the Anophelinae. Circles are colored by the class of the host with the highest proportion of associations. Most mosquitoes have a strong host preference towards a single class of vertebrate host, although there are exceptions, particularly in *Culex*.

Supplementary Figure 6

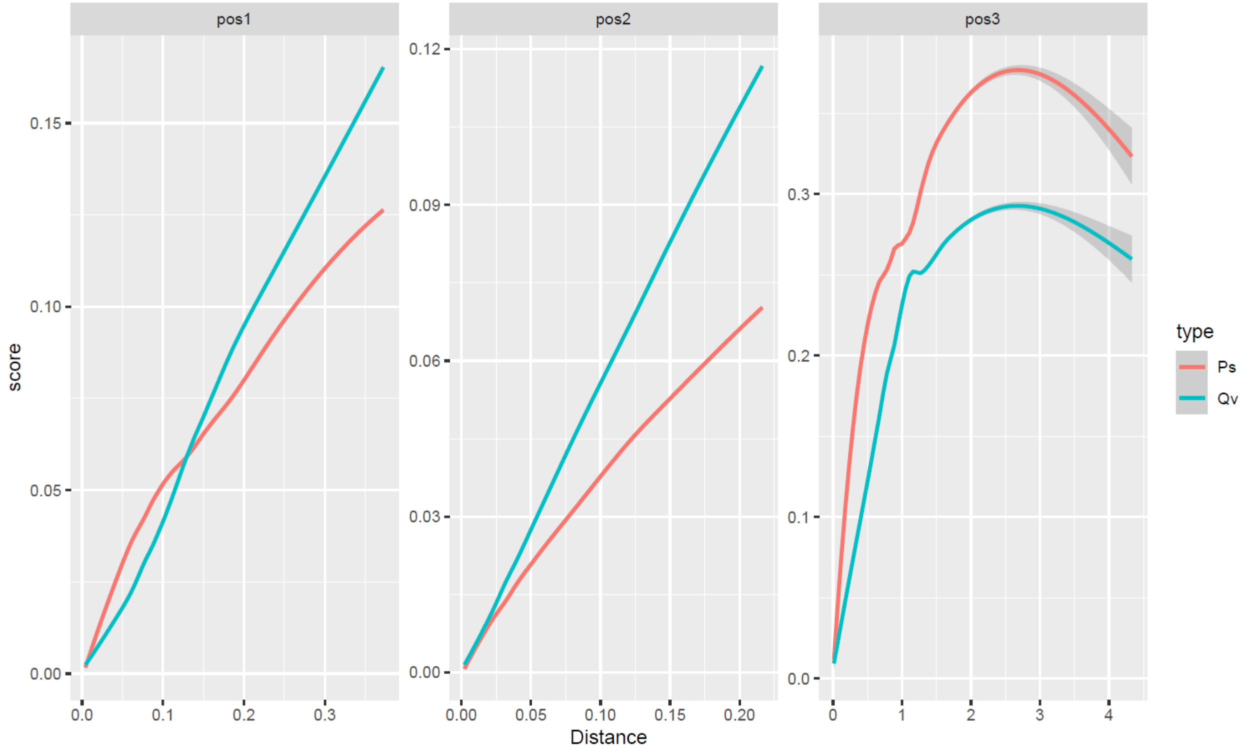

Saturation plots per codon position, with genetic distance on the X axis of each plot and transition (Ps) and transversion (Qv) ratios on the Y axis. Note difference in axis scales. Position 3 shows strong signs of saturation, with non-linear relationships between genetic distance and observed Ps/Qv ratios. Position 1 shows weak signs of saturation, as well. Regressions are local polynomial regression fits with 95% confidence intervals.

Supplementary Figure 7

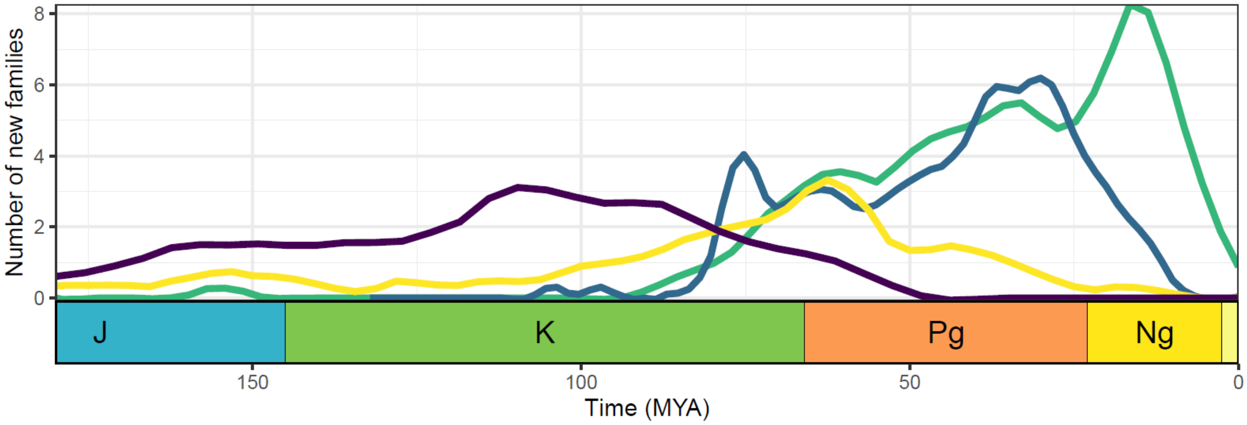

Family-level lineage through time plots for major vertebrate classes which are commonly fed on by mosquitoes: amphibians (purple), reptiles (yellow), birds (blue), mammals (green).

Supplementary Figure 8

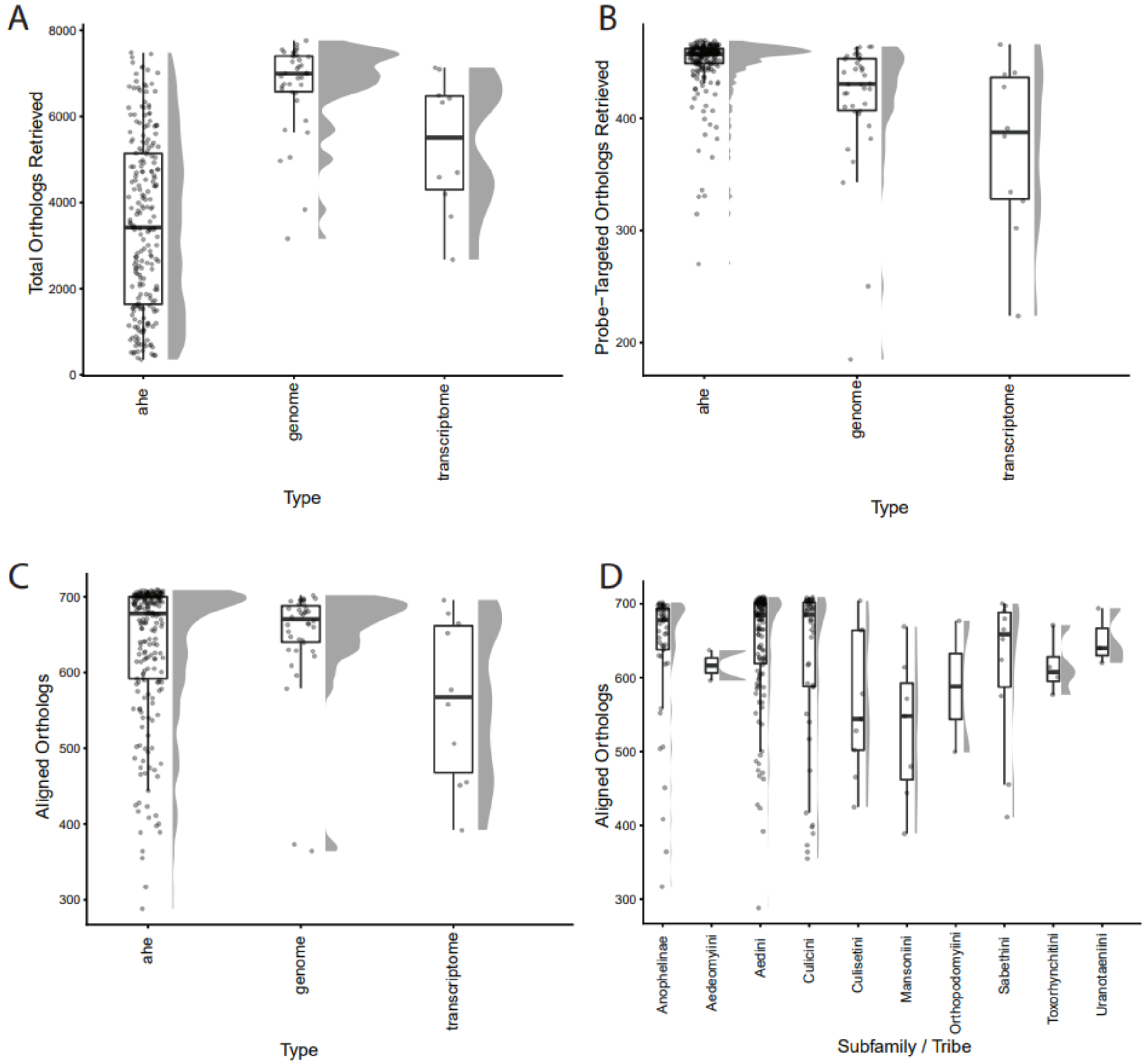

Ortholog recovery efficiency as raincloud plots, across genetic data of different types and across different taxonomic groupings of mosquitoes. Shaded areas indicate the density of observations, while boxplots show a thick black line for the median, a box showing the interquartile range, and whiskers that are 1.5 +/- the interquartile range. Genomes in general recovered more orthologs from the total Culicologs catalog (A), but recovery was similar when considered probe-targeted orthologs alone (B). Genomes and transcriptomes had similar numbers of orthologs in our primary alignment we analyzed (C), and different taxonomic groups of mosquitoes had similar ortholog recovery, although our relatively low sampling in the Mansoniini resulted in statistically different ortholog recoveries between this tribe and the Aedini (D).

Supplementary Figure 9

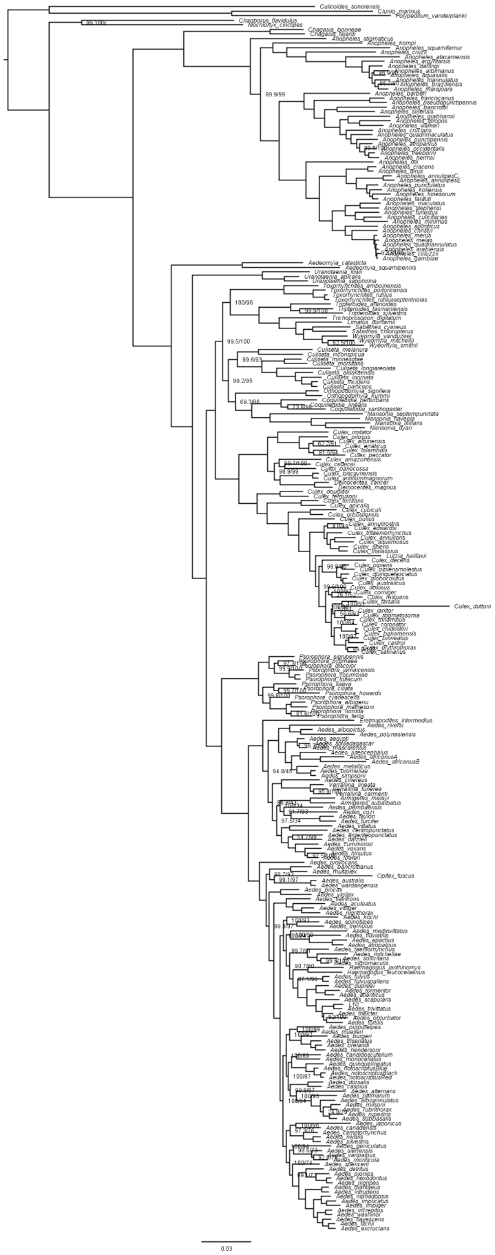

The maximum likelihood phylogeny inferred in IQ-Tree, based on the alignment of only orthologs targeted by AHE probes. Support values are SH-like aLRT branch support values and Ultrafast Bootstrap values, and only values below 100/100 are shown.

Supplementary Figure 10

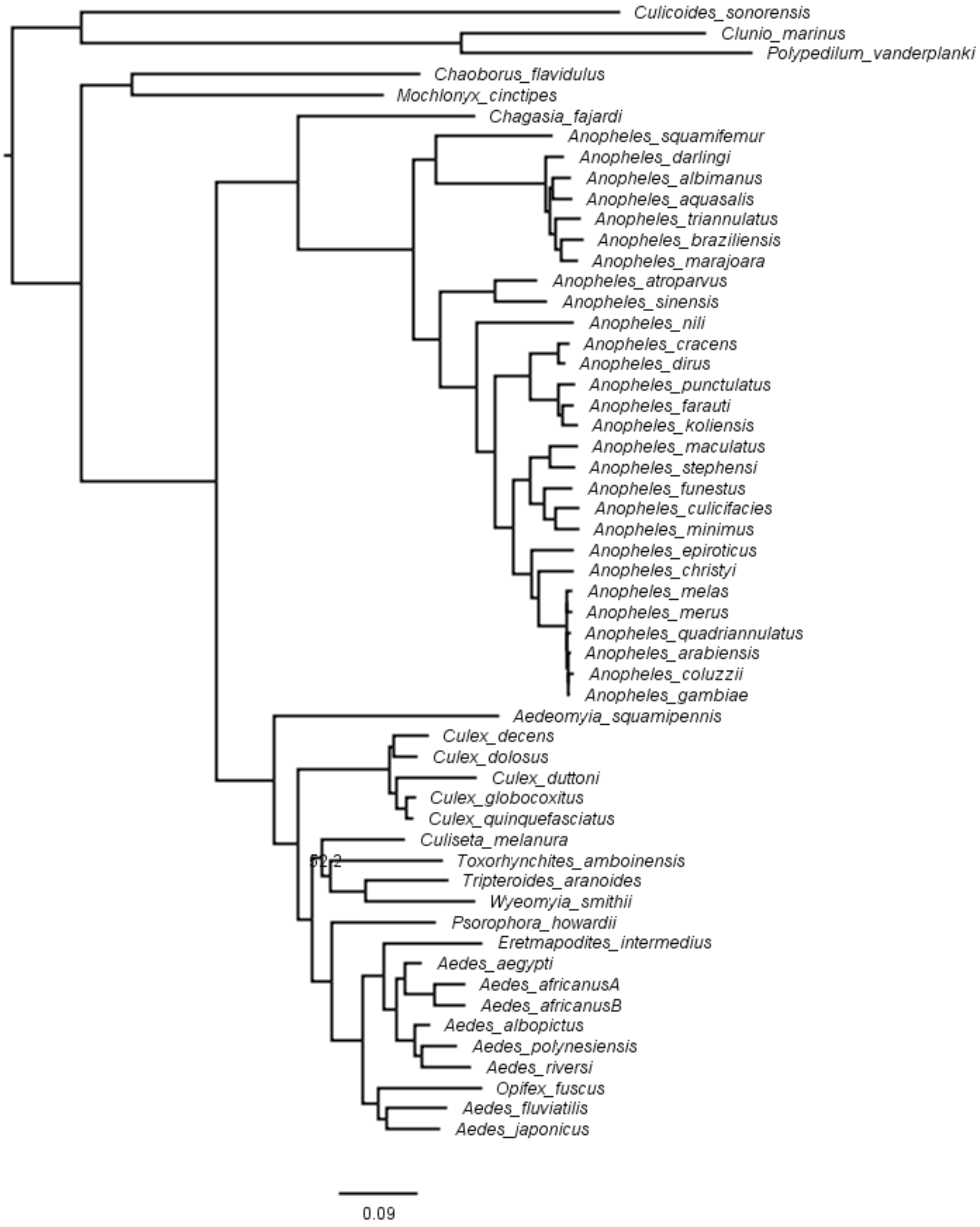

The maximum likelihood phylogeny inferred in IQ-Tree from genomes and transcriptomes. Support values are SH-like aLRT branch support values, and only values below 100 are shown.

Supplementary Figure 11

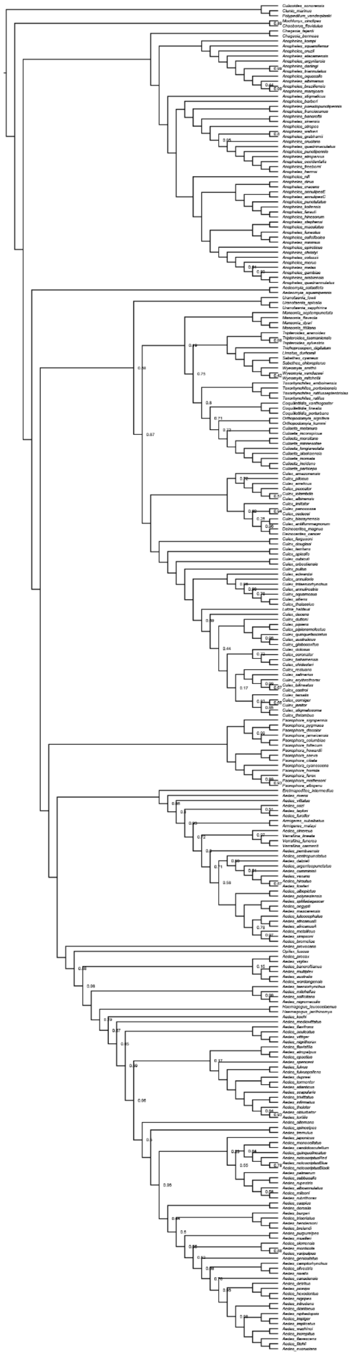

The cladogram inferred by ASTRAL. Support values are local posterior probabilities, and only values below 1 are shown. For easier visualization of relationships and support values, ASTRAL topologies are shown as cladograms.

Supplementary Figure 12

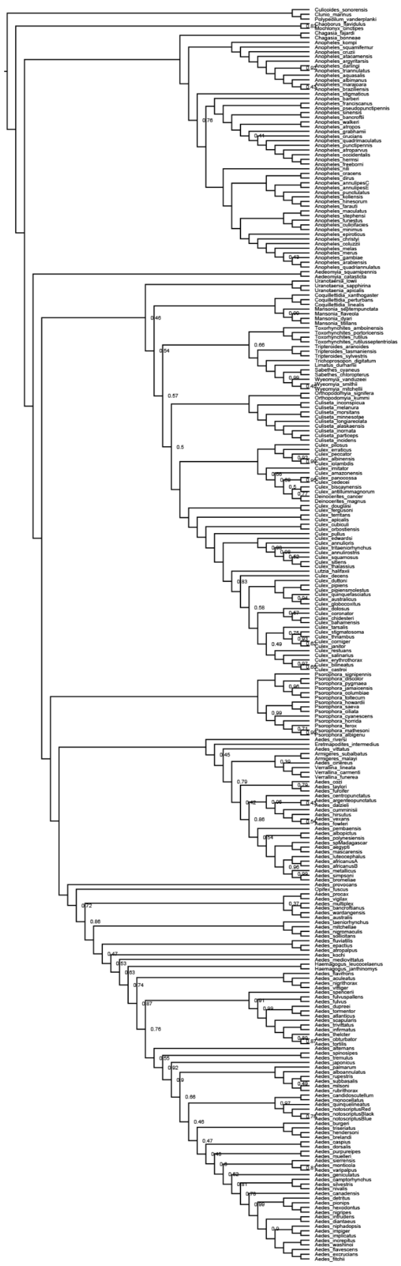

The cladogram inferred by ASTRAL, considering only gene trees with at least 214 species (85% of taxa in the analysis). Support values are local posterior probabilities, and only values below 1 are shown. For easier visualization of relationships and support values, ASTRAL topologies are shown as cladograms.

Supplementary Figure 13

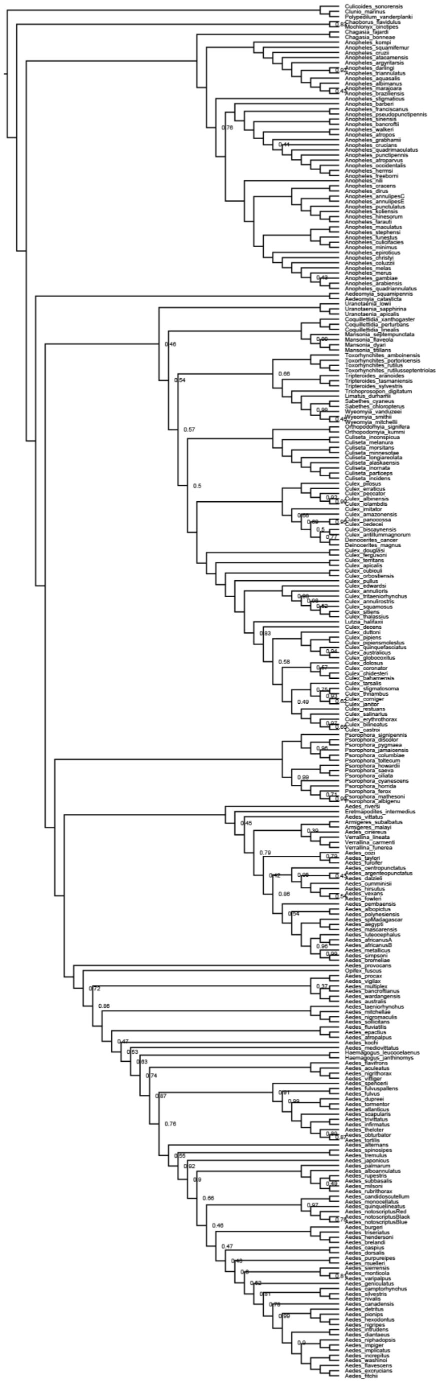

The cladogram inferred by ASTRAL, considering only gene trees with at least 241 species (90% of taxa in the analysis). Support values are local posterior probabilities, and only values below 1 are shown. For easier visualization of relationships and support values, ASTRAL topologies are shown as cladograms.

Supplementary Figure 14

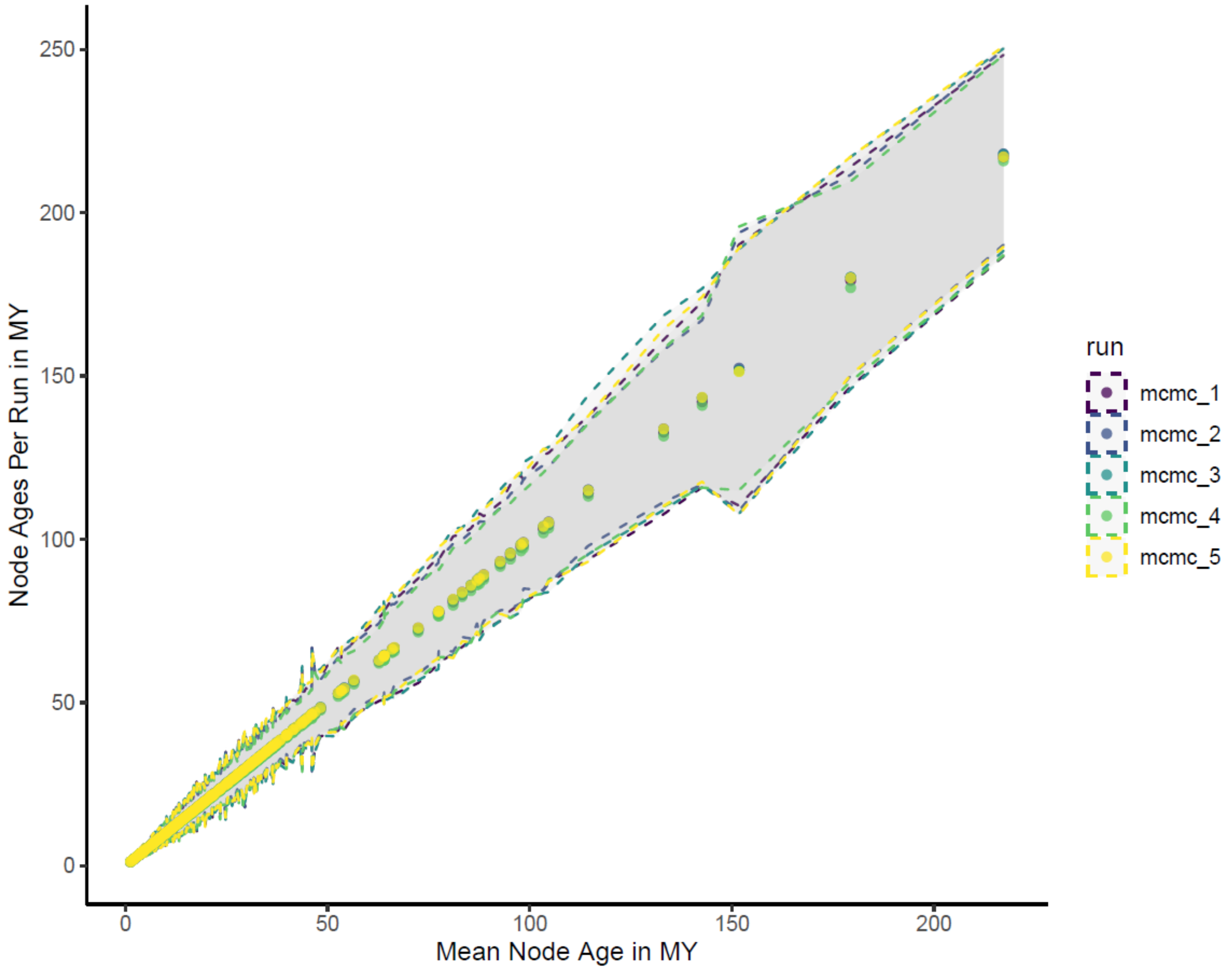

Five separate chains run in MCMCTree converge on similar node estimates and with overlapping 95% HPDs. Node estimates are points (given some opacity, so other points can be visualized where they overlap), while dashed lines are estimates of the 95% highest posterior density per node.

Supplementary Figure 15

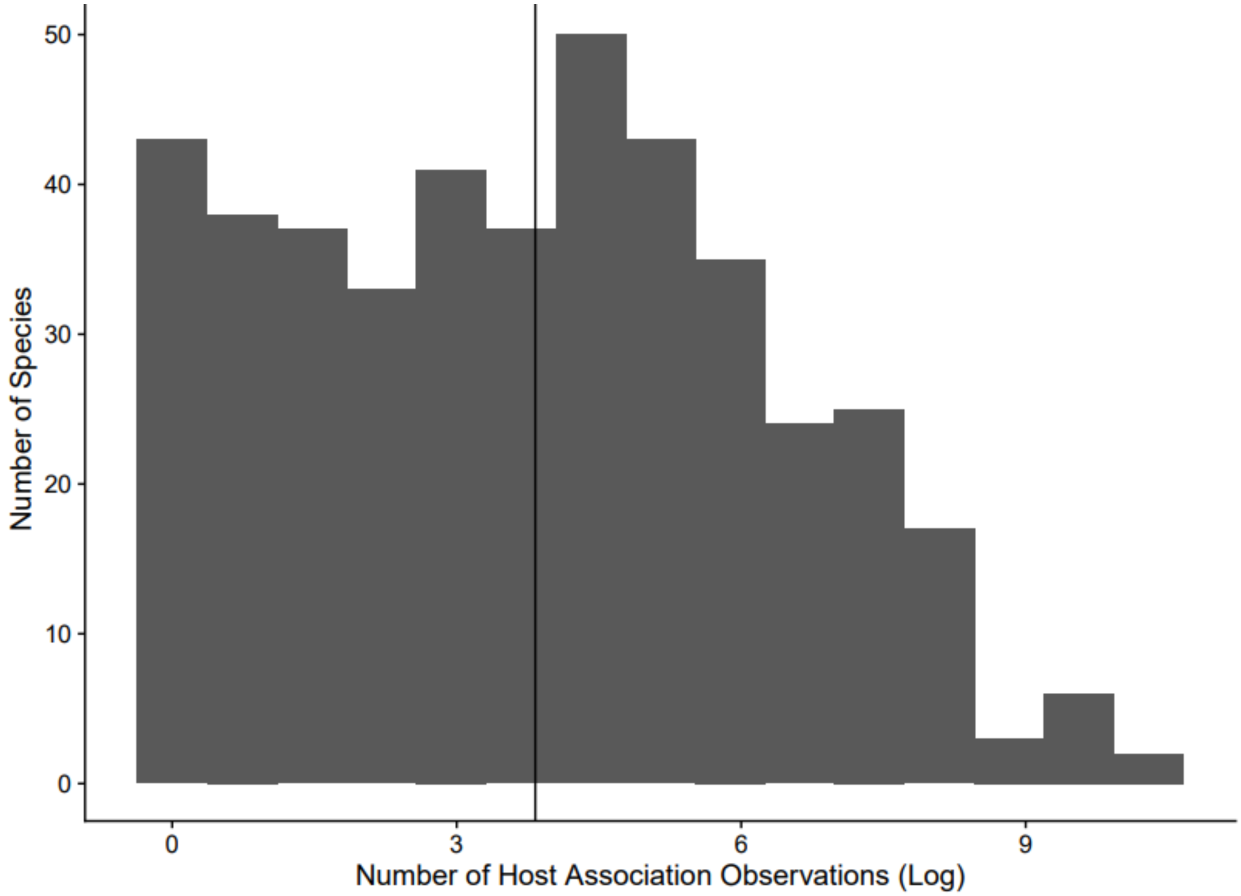

A histogram showing the distribution of host associations observed for mosquito species. Here, a single host association is one mosquito observed with a blood meal identifiable to a given class of host. Host associations shown on a log scale. Black horizontal line indicates the median number of host associations observed across species, 46.

Supplementary Figure 16

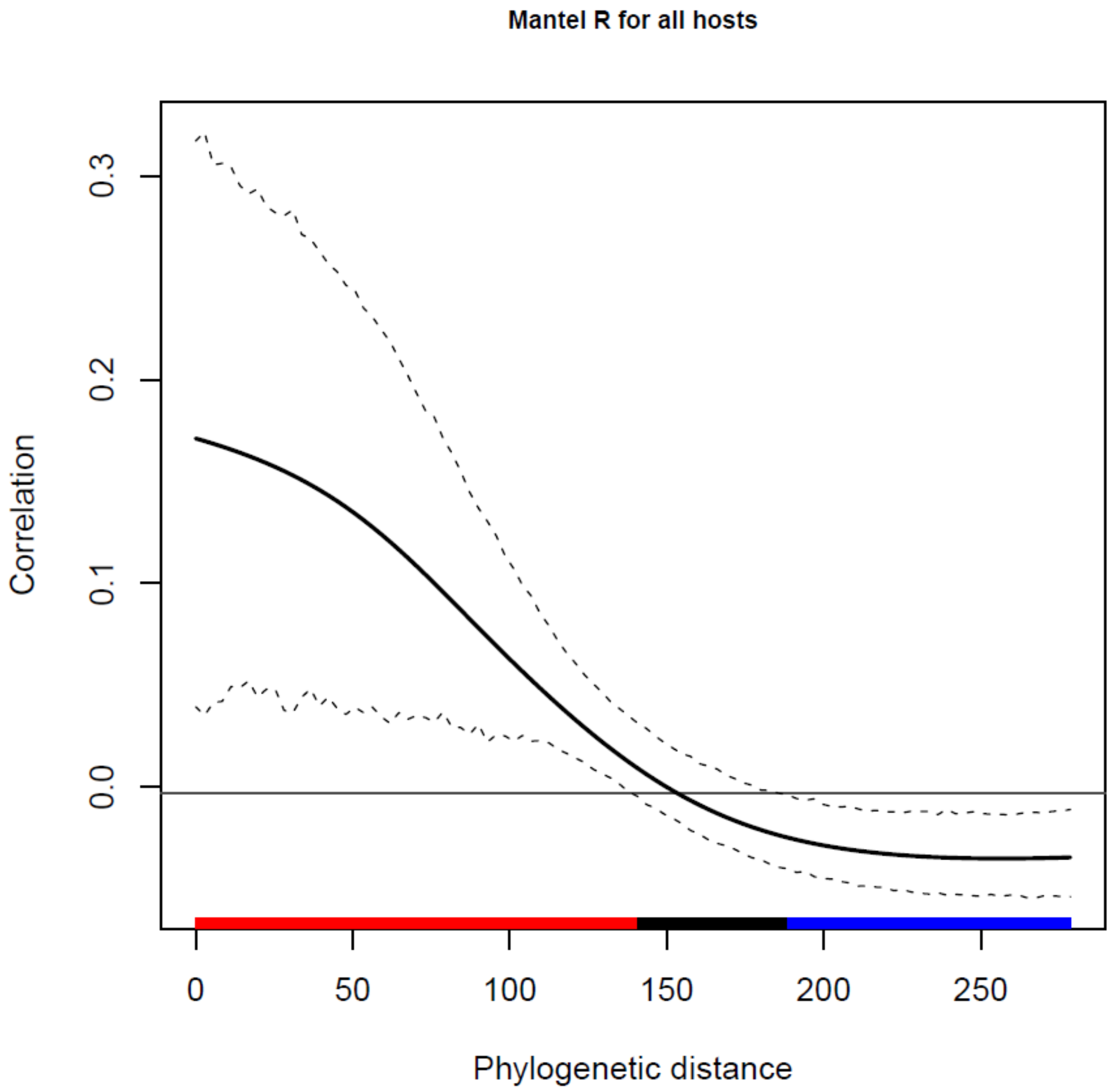

A phylogenetic correlogram showing the association between phylogenetic distance and host association for all classes of vertebrate hosts in our analysis, including Anopheline mosquitoes. There is a significant positive correlation at short to intermediate genetic distances, with a negative correlation at deep genetic distances. Dashed lines indicate upper and lower 95% confidence limits derived from 1000 bootstrap replicates.

Supplementary Figure 17

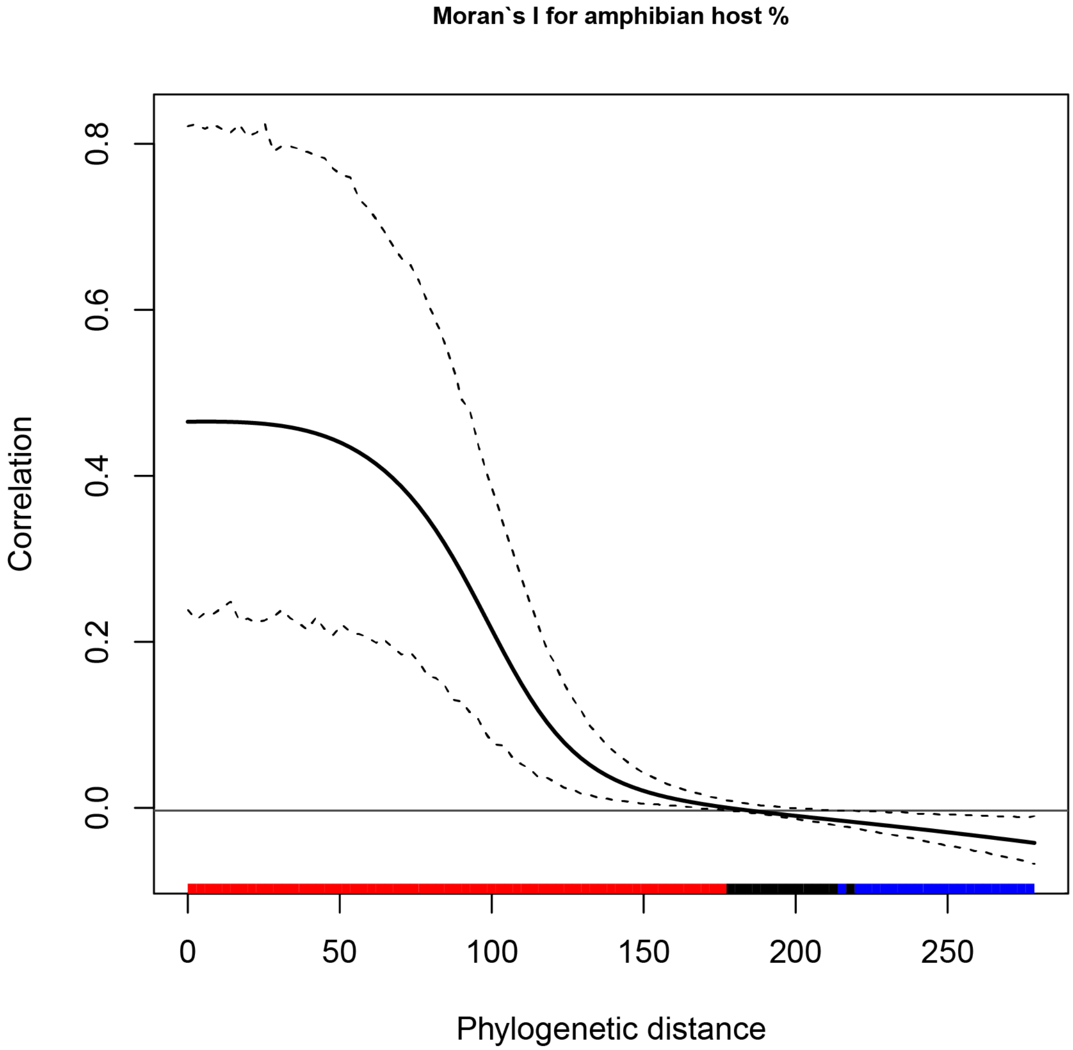

A phylogenetic correlogram showing the association between phylogenetic distance and host association with amphibians in our analysis. At short genetic distances, there is a positive correlation between genetic distance and proportion of amphibians fed upon, but no association at intermediate distances. Dashed lines indicate upper and lower 95% confidence limits derived from 1000 bootstrap replicates.

Supplementary Figure 18

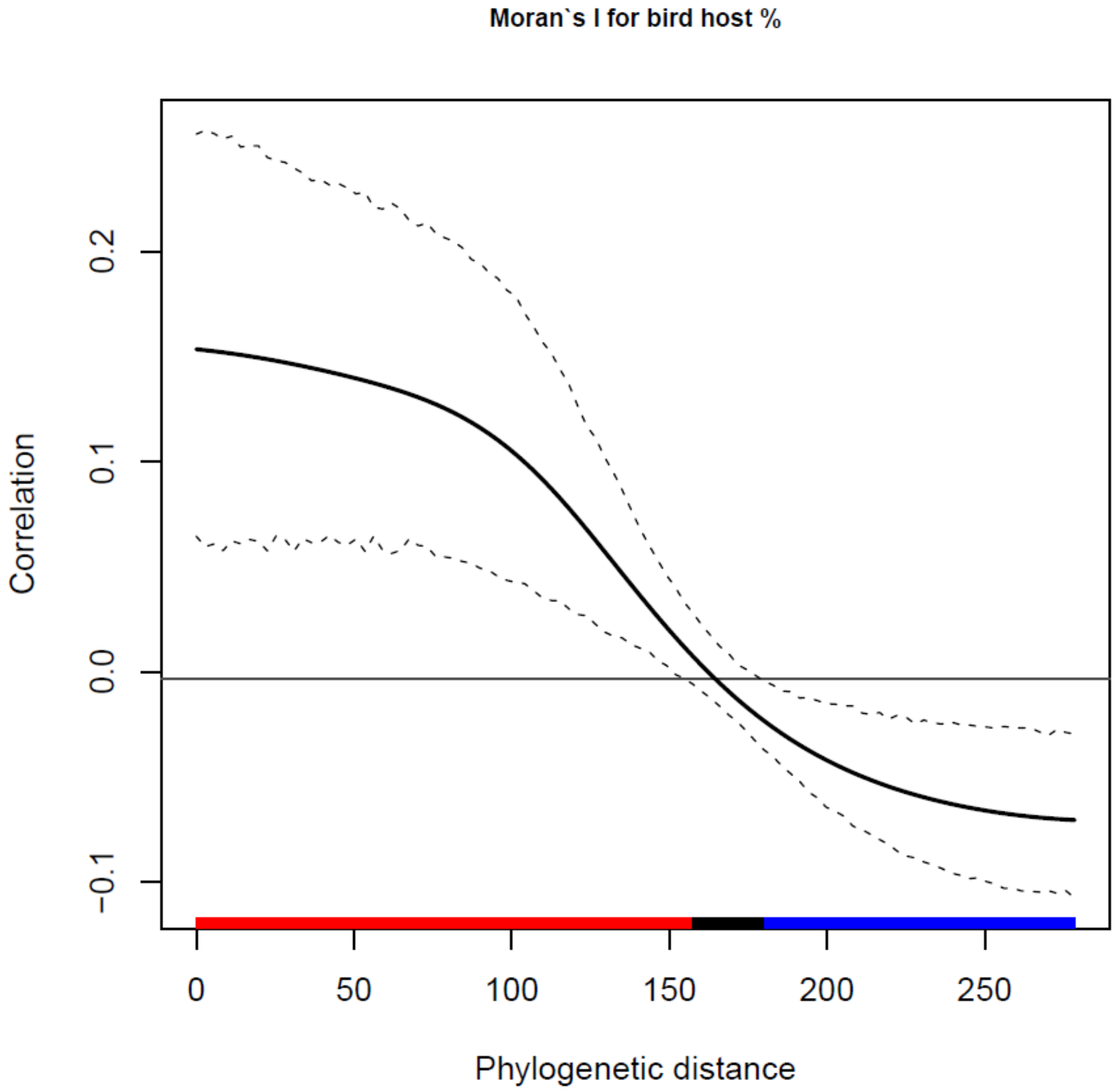

A phylogenetic correlogram showing the association between phylogenetic distance and host association with birds in our analysis. At short to intermediate genetic distances, there is a positive correlation between genetic distance and proportion of birds fed upon, with a negative correlation at deep genetic distances. Dashed lines indicate upper and lower 95% confidence limits derived from 1000 bootstrap replicates.

Supplementary Figure 19

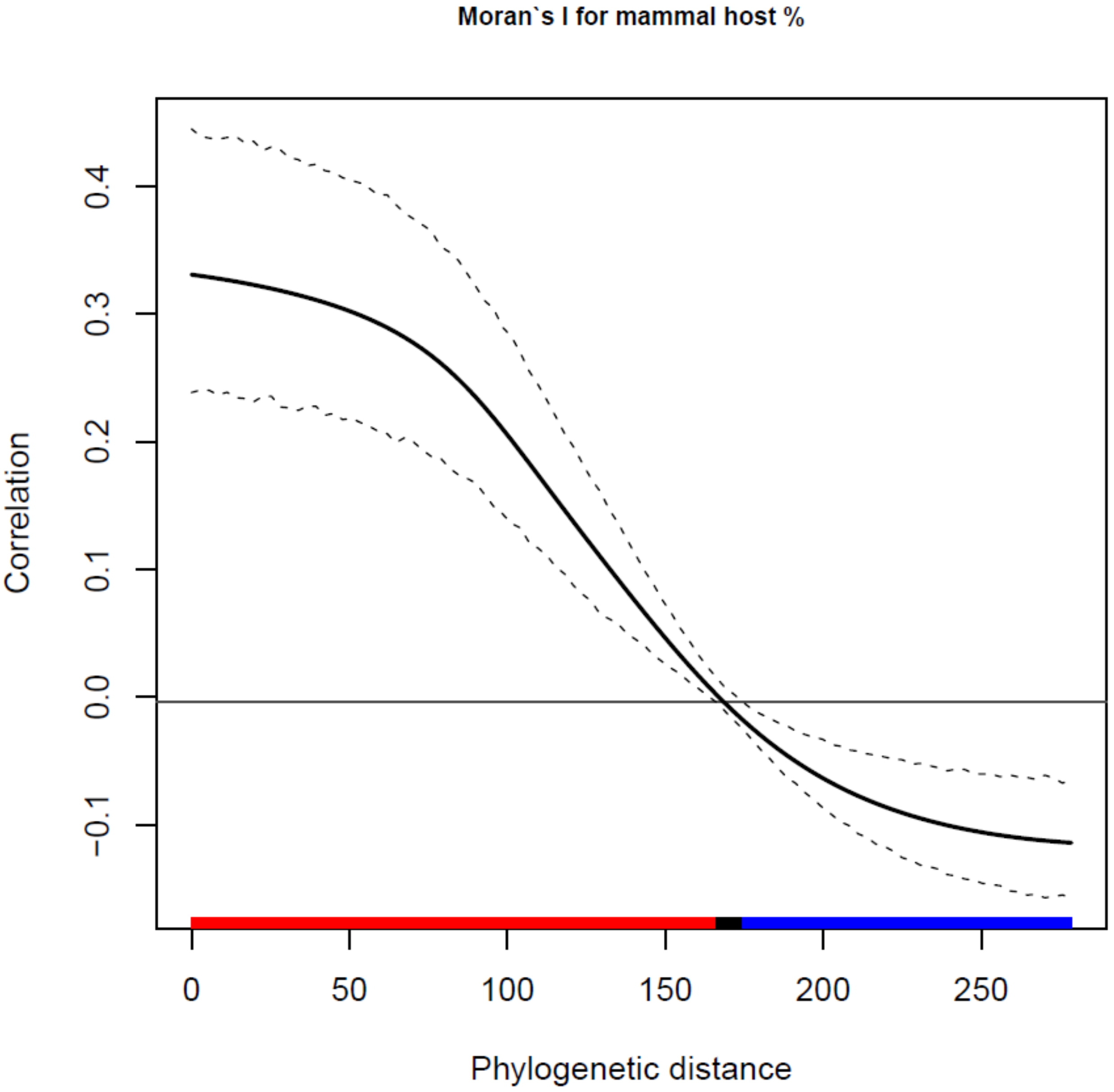

A phylogenetic correlogram showing the association between phylogenetic distance and host association with mammals. This is a similar pattern to other host classes - at short to intermediate genetic distances, there is a positive correlation between genetic distance and proportion of birds fed upon, with a negative correlation at deep genetic distances. Dashed lines indicate upper and lower 95% confidence limits derived from 1000 bootstrap replicates.

Supplementary Figure 20

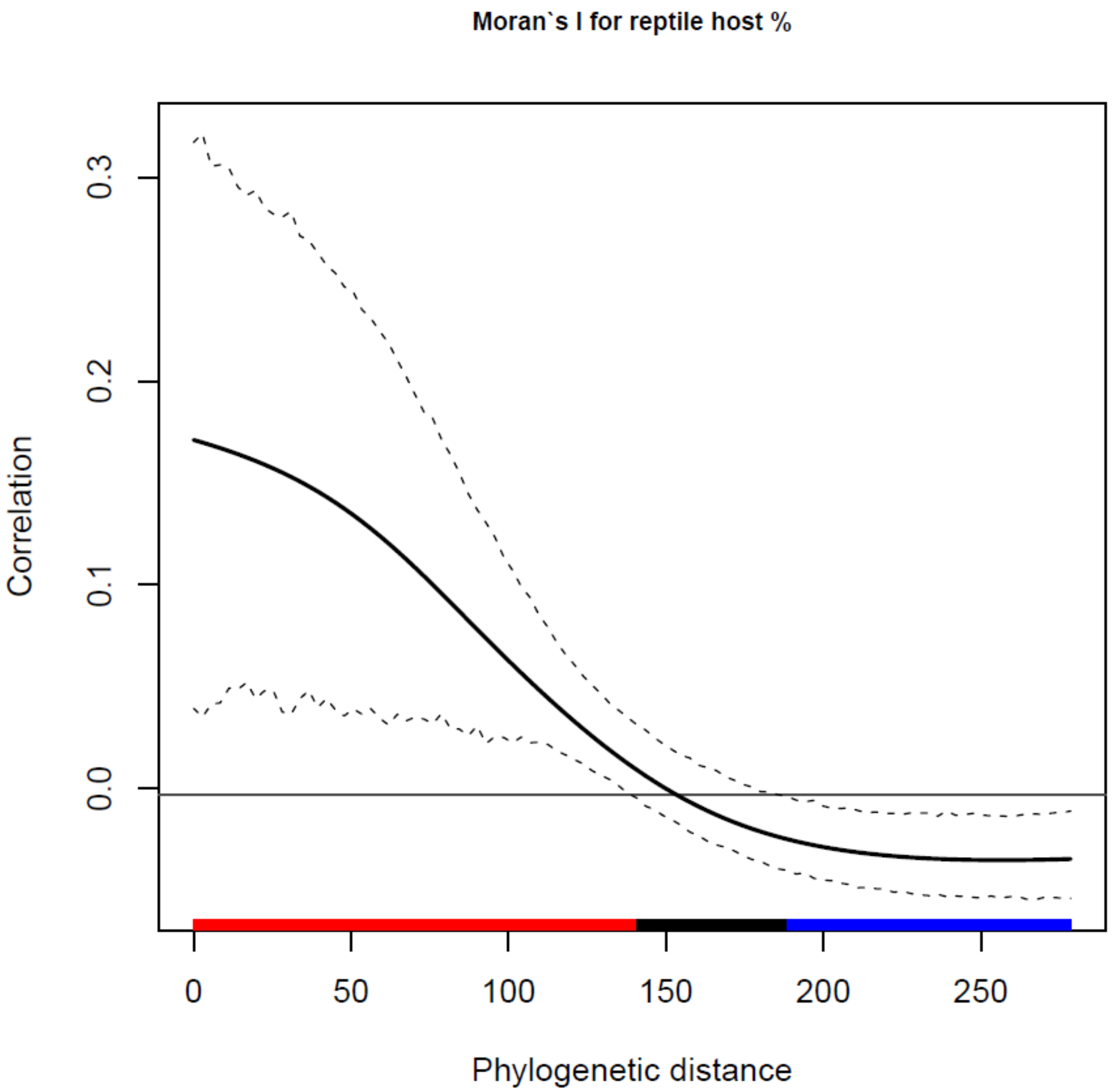

A phylogenetic correlogram showing the association between phylogenetic distance and host association with reptiles in our analysis. Dashed lines indicate upper and lower 95% confidence limits derived from 1000 bootstrap replicates.

Supplementary Figure 21

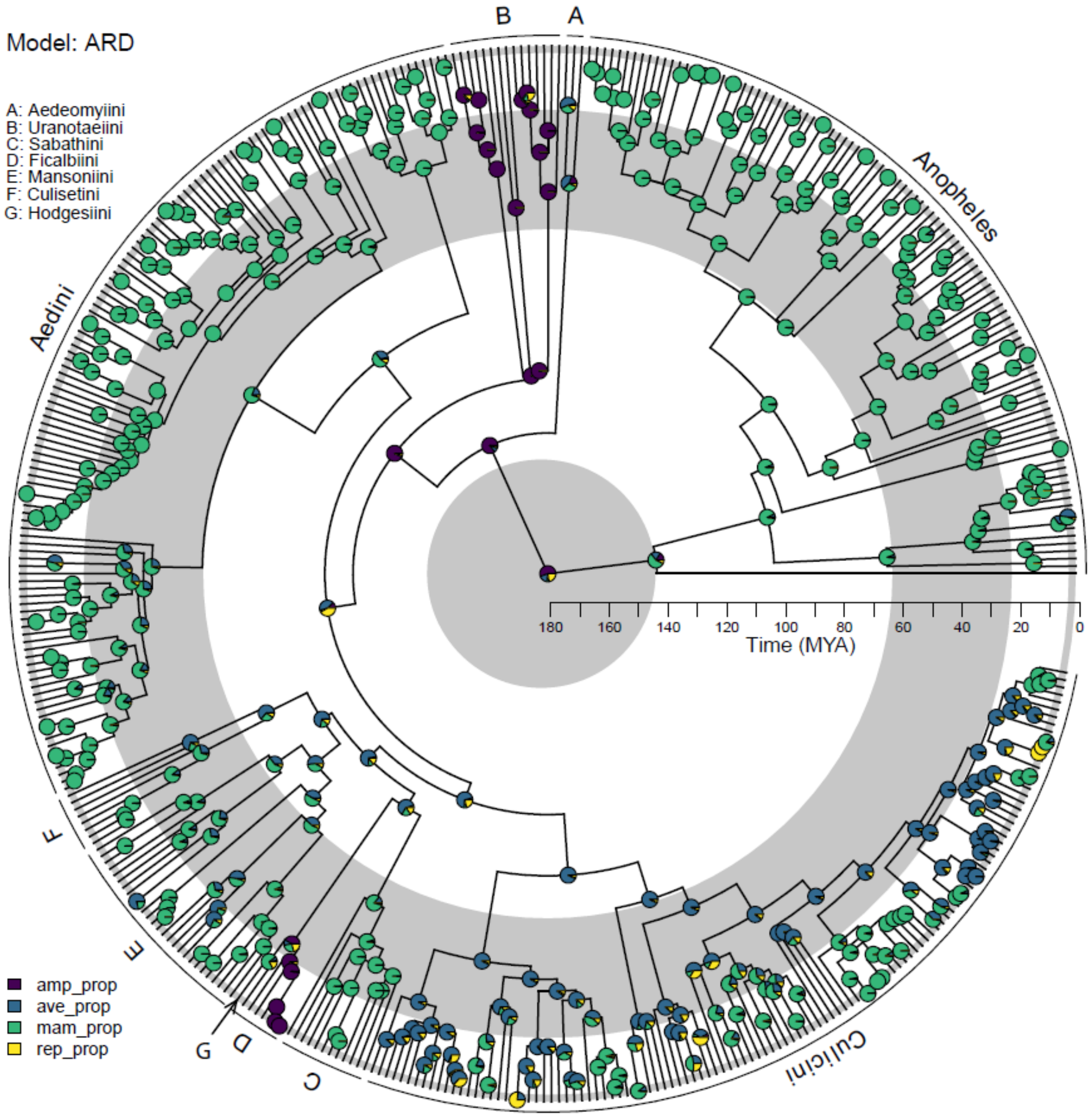

The ancestral state reconstruction that includes tribes added via TACT, Ficalbiini and Hodgesiini. As in our other ancestral state reconstructions, amphibian-associated is the most well-supported ancestral state.

Supplementary Figure 22

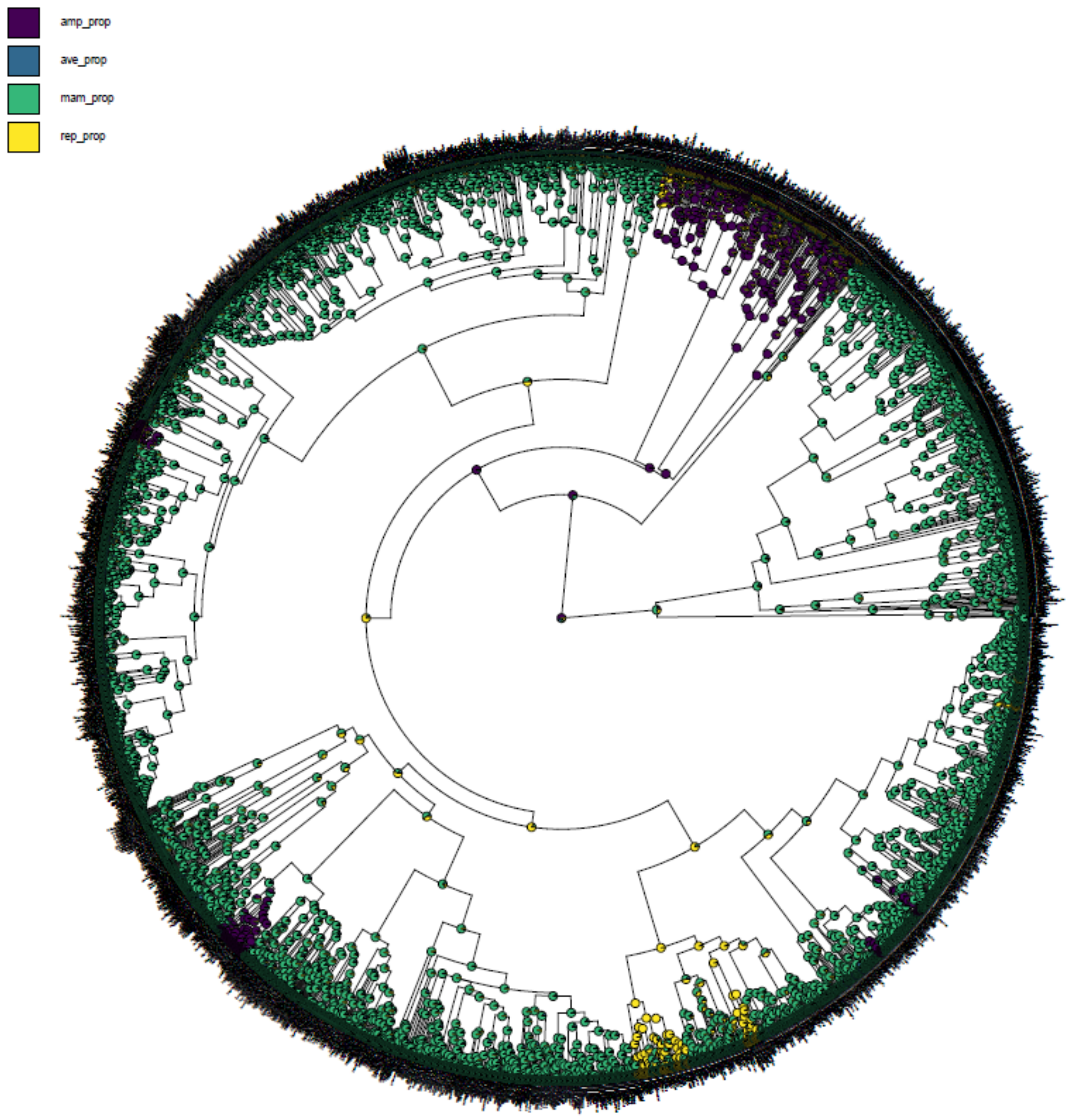

The ancestral state reconstruction that includes all mosquito species. For species without bloodhost information, a flat prior (equal probability of any feeding state) was supplied. As in our other ancestral state reconstructions, amphibian-associated is the most well-supported ancestral state.

Supplementary Figure 23

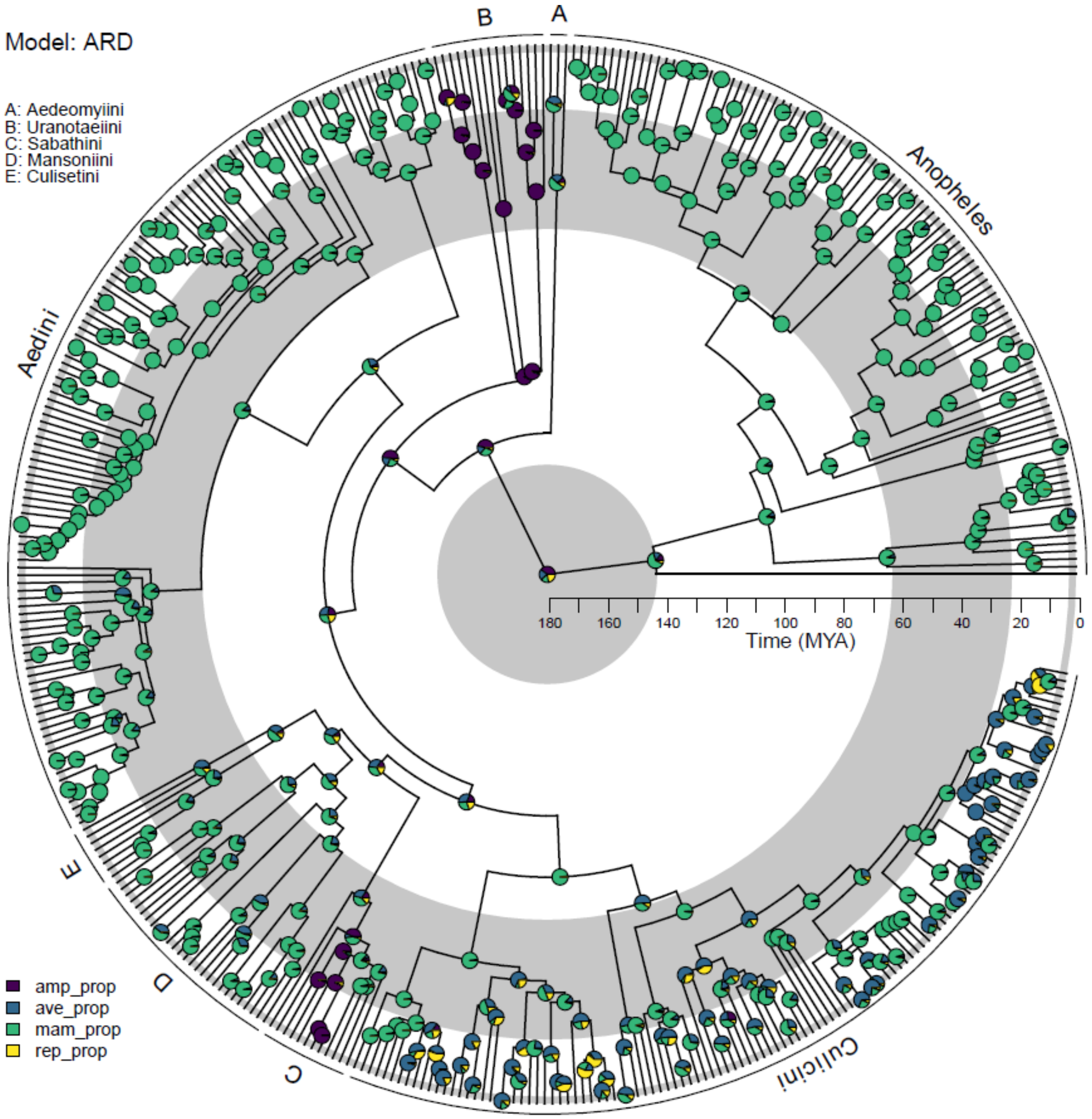

The summarized ancestral state reconstruction using posterior trees sampled from MCMCTree and fed into TACT to produce a range of topologies. This analysis used only data from sampled tribes. As in our other ancestral state reconstructions, amphibian-associated is the most well-supported ancestral state.
