# Supplementary material for: Phylogenomics reveals the history of host use in mosquitoes
Source: Nat Commun. 2023 Oct 6;14:6252. doi: 10.1038/s41467-023-41764-y (PMC10558525; doi:10.1038/s41467-023-41764-y)
Supplement: Supplementary file 3 — Reporting Summary [file 41467_2023_41764_MOESM3_ESM.pdf]

nature portfolio

Corresponding author(s): Brian M Wiegmann

Last updated by author(s): 2023 - 08- 07

Reporting Summary

Nature Portfolio wishes to improve the reproducibility of the work that we publish. This form provides structure for consistency and transparency in reporting. For further information on Nature Portfolio policies, see our Editorial Policies and the Editorial Policy Checklist.

Statistics

For all statistical analyses, confirm that the following items are present in the figure legend, table legend, main text, or Methods section.

n/a	Confirmed	
☐	☒	The exact sample size (*n*) for each experimental group/condition, given as a discrete number and unit of measurement
☒	☐	A statement on whether measurements were taken from distinct samples or whether the same sample was measured repeatedly
☒	☐	The statistical test(s) used AND whether they are one- or two-sided<br>*Only common tests should be described solely by name; describe more complex techniques in the Methods section.*
☒	☐	A description of all covariates tested
☐	☒	A description of any assumptions or corrections, such as tests of normality and adjustment for multiple comparisons
☐	☒	A full description of the statistical parameters including central tendency (e.g. means) or other basic estimates (e.g. regression coefficient) AND variation (e.g. standard deviation) or associated estimates of uncertainty (e.g. confidence intervals)
☒	☐	For null hypothesis testing, the test statistic (e.g. *F*, *t*, *r*) with confidence intervals, effect sizes, degrees of freedom and *P* value noted<br>*Give P values as exact values whenever suitable.*
☐	☒	For Bayesian analysis, information on the choice of priors and Markov chain Monte Carlo settings
☒	☐	For hierarchical and complex designs, identification of the appropriate level for tests and full reporting of outcomes
☒	☐	Estimates of effect sizes (e.g. Cohen's *d*, Pearson's *r*), indicating how they were calculated

Our web collection on statistics for biologists contains articles on many of the points above.

Software and code

Policy information about availability of computer code

Data collection

trimmomatic v 0.36
fastp v0.20
trinity v2.4
SPADES 3.0.0
GATB-Minia Pipeline v. release 4/1/2020 (https://github.com/GATB/minia)
AUGUSTUS v.3.4 (github.com/Gaius-Augustus/Augustus
Orthograph v.0.7.1 https://github.com/mptrsen/Orthograph)
OMA v 2.4.1 https://omabrowser.org/oma/home/
Google Scholar

Data analysis

Culicitree github page (https://github.com/jsoghigian/culicitree)
BLAST https://blast.ncbi.nlm.nih.gov/
DAMBE7 http://dambe.bio.uottawa.ca/DAMBE/dambe.
R v4.1
ggplot2 3.4.1
IQTree 1.4.2
ape 5.4.1
FigTree v 1.4.4
ASTRAL -II
MCMCTree (http://abacus.gene.ucl.ac.uk/software/paml.html)
mcmc3r (https://github.com/dosreislab/mcmc3r)

ggtree v3.2 (https://guangchuangyu.github.io/software/ggtree/)
deeptime v0.2.3(https://github.com/deeptime-ml/deeptime)
TACT v0.4.1 -Taxonomy Aided Complete Trees (https://pypi.org/project/tact/)
phanghorn v2.11 (https://github.com/KlausVigo/phangorn)
geomorph ver. 4.0.1
phyloSignal ver 1.3
phytools package ver 1.0-1

For manuscripts utilizing custom algorithms or software that are central to the research but not yet described in published literature, software must be made available to editors and reviewers. We strongly encourage code deposition in a community repository (e.g. GitHub). See the Nature Portfolio guidelines for submitting code & software for further information.

Data

Policy information about availability of data

All manuscripts must include a data availability statement. This statement should provide the following information, where applicable:

- Accession codes, unique identifiers, or web links for publicly available datasets
- A description of any restrictions on data availability
- For clinical datasets or third party data, please ensure that the statement adheres to our policy

The nucleotide sequence data reported herein are archived in the NCBI, NIH SRA (Sequence Read Archive) under SRA Bioproject Number PRJNA907815. The phylogenetic trees generated in this study are available on our GitHub (https://github.com/jsoghigian/culicitree), DOI:10.5281/zenodo.8212811. Nucleotide and amino acid alignments we analyzed, along with input files we used for analyses conducted in R, are available on Figshare, DOI: 10.6084/m9.figshare.23826144. We used publicly available genomes and transcriptomes for ortholog catalog creation, summarized here: Anopheles (Cellia) gambiae (AgamP4.11, [https://vectorbase.org/vectorbase/app/record/dataset/TMPTX_agamPimperena]), An. (Anopheles) atroparvus (AatrE3.1, https://vectorbase.org/vectorbase/app/record/dataset/TMPTX_aatrEBRO]), An. (Nyssorynchus) albimanus (AalbS2.6, [https://vectorbase.org/vectorbase/app/record/dataset/TMPTX_aalbSTECLA]), An. (Lophophodomyia) squamifemur (Asqu1.1, which we assembled early in the project), Aedes aegypti (AaegL5.1, [https://vectorbase.org/vectorbase/app/record/dataset/TMPTX_aaegLVP_AGWG]) and Aedes albopictus (AaloF1.2, [https://vectorbase.org/vectorbase/app/record/dataset/TMPTX_aalbFoshan]), Culex quinquefasciatus (CpipJ2_4, [https://vectorbase.org/vectorbase/app/record/dataset/TMPTX_cquiJohannesburg]), and two transcriptomes from Toxorynchites amboinensis ([https://figshare.com/articles/dataset/Sequence_and_functional_annotation_of_T_amboinensis_genes/1092617]) and Tripteroides aranoides (GGBM00000000.1, [https://www.ncbi.nlm.nih.gov/nuccore/GGBM00000000.1]). The mosquito bloodmeal data we analyzed is provided as Supplementary Data 4. Specimen data, including collection locale or relevant publication, is available in Supplementary Data 2.

Human research participants

Policy information about studies involving human research participants and Sex and Gender in Research.

Reporting on sex and gender	NA
Population characteristics	NA
Recruitment	NA
Ethics oversight	NA

Note that full information on the approval of the study protocol must also be provided in the manuscript.

Field-specific reporting

Please select the one below that is the best fit for your research. If you are not sure, read the appropriate sections before making your selection.

☐ Life sciences ☐ Behavioural & social sciences ☒ Ecological, evolutionary & environmental sciences

For a reference copy of the document with all sections, see nature.com/documents/nr-reporting-summary-flat.pdf

Ecological, evolutionary & environmental sciences study design

All studies must disclose on these points even when the disclosure is negative.

Study description	Phylogenomic analysis of orthologous nuclear gene loci obtained by anchored hybrid enrichment (AHE, exon capture) are used to reconstruct phylogenetic relationships among diverse species of mosquitoes (Diptera:Culicidae). The phylogenetic data, tree estimates, and biogeographic, fossil and bloodhost records are used to test hypotheses about the timing, distribution and historical pattern of host use specialization in the family.
Research sample	Mosquito specimens from collaborating laboratories and natural history collections were used to extract genomic DNA form indifividual specimens for anchored hybridization and high throughput sequencing. Nuclear gene orthologs were identified bioinformatically through comparison using published gene models and public databases. All blood-host information was taken from the published scientific literature.

Sampling strategy	Multiple species were selected to represent each established mosquito tribe in the classification. In large genera containing multiple human disease vectoring species.
Data collection	Data were collected by DNA extraction, library preparation, anchored enrichment and DNA sequencing using HiSeq4000 or NovaSeq6000 at the NC State University Genomic Sciences Laboratory. These procedures were carried by Brian K. Cassel, Charles Sither and the NCSU Genome Sciences laboratory.
Timing and spatial scale	Data were collected from individual mosquito specimens routinely and without time dependency in specific laboratory and sequencing experiments in 2019, 2020 and 2021.
Data exclusions	Only data that were considered to be low - yield DNA quality from DNA capture experiments were excluded from analysis.
Reproducibility	Multiple species were included in which gene capture efficacy and repoducibility can be verified in comparison against public genomic and transcriptomic data (e.g., Aedes aegypti, Anopheles gambiae)
Randomization	Phylogenetic analysis using maximum likelihood or Bayesian analysis uses random selection of taxon addition and intitial tree-building to avoid bias in tree search pathways.
Blinding	Blinding is not relevant in analysis of existing genetic variation for phylogenetic purposes.

Did the study involve field work? ☐ Yes ☒ No

Reporting for specific materials, systems and methods

We require information from authors about some types of materials, experimental systems and methods used in many studies. Here, indicate whether each material, system or method listed is relevant to your study. If you are not sure if a list item applies to your research, read the appropriate section before selecting a response.

Materials & experimental systems

n/a	Involved in the study	
☒	☐	Antibodies
☒	☐	Eukaryotic cell lines
☒	☐	Palaeontology and archaeology
☒	☐	Animals and other organisms
☒	☐	Clinical data
☒	☐	Dual use research of concern

Methods

n/a	Involved in the study	
☒	☐	ChIP-seq
☒	☐	Flow cytometry
☒	☐	MRI-based neuroimaging
