# Supplementary material for: Phylogenomics reveals the history of host use in mosquitoes
Source: Nat Commun. 2023 Oct 6;14:6252. doi: 10.1038/s41467-023-41764-y (PMC10558525; doi:10.1038/s41467-023-41764-y)
Supplement: Supplementary file 4 — Description of Additional Supplementary Files [file 41467_2023_41764_MOESM4_ESM.pdf]

Description of Additional Supplementary Files Document

Supplementary Data 1

Description: The list of species used in our analyses, with bioregion where mosquitoes we analysed are found.

Supplementary Data 2

Description: The details on where particular specimens were collected or data was retrieved from, as well as sequencing platform and data source type (e.g., genomes or transcriptomes or anchor hybrid enrichment).

Supplementary Data 3

Description: Mosquito taxonomy information.

Supplementary Data 4

Description: Mosquito bloodmeal information analysed in this study.
